# mRNA-miRNA-lncRNA Regulatory Network in Nonalcoholic Fatty Liver Disease

**DOI:** 10.3390/ijms22136770

**Published:** 2021-06-24

**Authors:** Marwa Matboli, Shaimaa H. Gadallah, Wafaa M. Rashed, Amany Helmy Hasanin, Nada Essawy, Hala M. Ghanem, Sanaa Eissa

**Affiliations:** 1 Department of Medical Biochemistry and Molecular Biology, Faculty of Medicine, Ain Shams University, Cairo 11382, Egypt; 2 Department of Biochemistry, Faculty of Science, Ain Shams University, Cairo 11382, Egypt; shaimaahamady@sci.asu.edu.eg (S.H.G.); halaghanem@sci.asu.edu.eg (H.M.G.); 3 Department of Research, Children’s Cancer Hospital-57357, Cairo 11382, Egypt; wafaaanor@gmail.com; 4 Department of Clinical Pharmacology, Faculty of Medicine, Ain Shams University, Cairo 11382, Egypt; amany_hasanin@med.asu.edu.eg; 5 Institut Pasteur, CEDEX 15, 75724 Paris, France; nada.essawy@pasteur.fr

**Keywords:** NAFLD, Non-Alcoholic SteatoHepatitis (NASH), Hippo signaling, miRNAs, lncRNAs, ncRNAs network

## Abstract

Aim: we aimed to construct a bioinformatics-based co-regulatory network of mRNAs and non coding RNAs (ncRNAs), which is implicated in the pathogenesis of non-alcoholic fatty liver disease (NAFLD), followed by its validation in a NAFLD animal model. Materials and Methods: The mRNAs–miRNAs–lncRNAs regulatory network involved in NAFLD was retrieved and constructed utilizing bioinformatics tools. Then, we validated this network using an NAFLD animal model, high sucrose and high fat diet (HSHF)-fed rats. Finally, the expression level of the network players was assessed in the liver tissues using reverse transcriptase real-time polymerase chain reaction. Results: in-silico constructed network revealed six mRNAs (*YAP1, FOXA2, AMOTL2, TEAD2, SMAD4* and *NF2*), two miRNAs (*miR-650* and *miR-1205*), and two lncRNAs (RPARP-AS1 and *SRD5A3-AS1*) that play important roles as a co-regulatory network in NAFLD pathogenesis. Moreover, the expression level of these constructed network–players was significantly different between NAFLD and normal control. Conclusion and future perspectives: this study provides new insight into the molecular mechanism of NAFLD pathogenesis and valuable clues for the potential use of the constructed RNA network in effective diagnostic or management strategies of NAFLD.

## 1. Introduction

Non-alcoholic fatty liver disease (NAFLD) is one of the common chronic liver diseases [1] with a global prevalence of ~25% [2]. In Egypt, it was reported in a cross-sectional study that NAFLD prevalence among children and adolescents was 15.8% [3]. NAFLD includes a spectrum of disorders ranging from liver simple steatosis (SS) to non-alcoholic steatohepatitis (NASH) that further increases the risk of developing cirrhosis and hepatocellular carcinoma [4]. Usually, NAFLD coexists with metabolic disorders, including obesity, type 2 diabetes (T2DM) and cardiovascular disease (CVD). The presence of NAFLD increases the incidence of T2DM while diabetes aggravates NAFLD to more severe disorders [5]. Moreover, NAFLD patients with concomitant T2DM are at highest risk for CVD [6]. Therefore, recent studies discussed the predictive role of NAFLD and liver fibrosis in both T2DM and CVD development [7,8].

The development of NAFLD is the result of a combination of genetic, environmental and metabolic factors [9]. Moreover, the pathogenesis of NAFLD is not yet entirely understood and the mechanism of how the simple steatosis of fatty liver progresses to NASH appears multifactorial. As a result, there have been multiple hypotheses describing the pathogenesis of NAFLD. The most widely accepted hypotheses are the two and three hits theories, which attribute NAFLD pathogenesis to inadequate hepatocyte proliferation and abnormal diffrentiation of hepatic progenitor cells to more unfavorable outcomes such as hepatic stellate cell (HSC) activation and liver fibrosis [10].

Recent emerging data showed that the molecular dysregulation of the Hippo pathway also contributes to the pathogenesis of NAFLD [11]. The Hippo pathway is a highly conserved master regulator of tissue regeneration and organ size by controlling several key cellular processes such as proliferation, viability, and differentiation [12].

Moreover, previous studies on non-coding ncRNAs, including miRNAs [13,14,15,16] and lncRNAs [17,18,19,20], have highlighted that co-regulation and interactions between these ncRNAs may clarify the molecular regulation and its complexity in NAFLD progression. The co-regulatory networks of miRNAs and lncRNAs in NAFLD pathophysiology may offer new early diagnostic biomarkers and therapeutic strategies [21]. 

Bioinformatic analysis plays a significant role in screening candidate biomarkers for various diseases [22]. Clearly, Gene Expression Omnibus (GEO) [23], an international public repository for functional genomic datasets, provides tools to help users identify, analyze, and visualize data relevant to discovering highly reliable biomarkers to human diseases [24]. In addition, functional annotation databases, e.g., the gene ontology (GO) and pathway databases, are significantly enriched in known target genes and can be used as fingerprints to identify genes relevant to some specific biological functions or diseases or to discover new potential drug targets [25]. In drug discovery, bioinformaticians can use high-throughput molecular data to (1) connect disease symptoms to genetic mutations, epigenetic modifications, and other environmental factors modulating gene expression, (2) identify drug targets that can either restore cellular function or eliminate malfunctioning cells, and (3) predict or refine drug candidates that can act upon the drug target to achieve the designed therapeutic result and minimize side effects [26].

Therefore, we aimed to construct “mRNAs—miRNAs—lncRNAs” regulatory network implicated in NAFLD pathogenesis based on in-silico analysis of microarray databases followed by its experimental validation in an NAFLD animal model.

## 2. Results

### 2.1. Co-Regulatory Network Construction

#### 2.1.1. Identification of Differentially Expressed mRNAs

**Figure 1:**
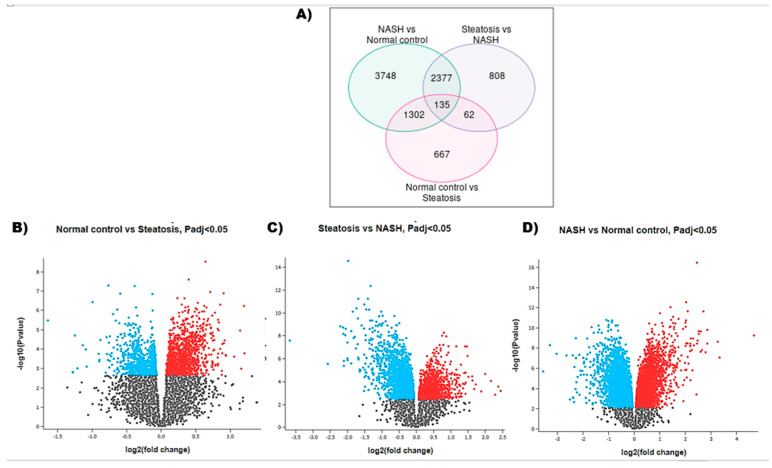
DEGs in GSE33814 dataset. (**A**) Venn diagram for DEGs between the three comparison groups. (**B**–**D**) Volcano plots of differentially expressed mRNAs between the three comparison groups. Red dots represent upregulated genes, blue dots represent downregulated genes, and black dots represent insignificantly differentially expressed genes, Padj < 0.05. DEG, differentially expressed gene; NASH, non-alcoholic steatohepatitis.

By normalization and analysis of the microarray dataset, a number of differentially expressed genes (DEGs) were identified in GSE33814 (Supplementary Table S1). The GSE33814 dataset contained 19,426 genes, among which 8664 DEGs were identified based on the cut-off criteria. The DEGs from the dataset between the groups were overlapped in a Venn diagram. A total of 135 DEGs overlapped in the normal control vs. steatosis, steatosis vs. NASH, and NASH vs. normal control groups, which may be involved in and associated to disease progression (Figure 1A). Additionally, analysis of the DEGs led to the identification of 1650 mRNAs between normal control and steatosis groups (including 1118 up-regulated and 532 down-regulated mRNAs), 2814 mRNAs between steatosis and NASH groups (including 1008 up-regulated and 1806 down-regulated mRNAs), and 6223 mRNAs between NASH and normal control groups (including 3164 up-regulated and 3059 down-regulated mRNAs). The up- and down-regulated DEGs between the three comparison groups were mapped on volcano plots to visualize their expression (Figure 1B–D).

#### 2.1.2. GO and Pathway Enrichment Analyses of DEGs

For functional enrichment analysis, 8664 DEGs between NASH, steatosis and normal groups were uploaded to the Enrichr database. The GO analysis was performed on biological level to retrieve the gene sets related to cell proliferation and differentiation while Kyoto Encyclopedia of Genes and Genomes (KEGG) and Reactome analyses were used for the retrieval of the Hippo signaling pathway-related gene set (Table 1). Finally, three integrated gene sets were obtained from enrichment analyses of DEGs (Table 2). The first set included genes related to cell proliferation (371 genes), the second included genes related to cell differentiation (148 genes), while the third set included genes related to the Hippo signaling pathway (86 genes). 

#### 2.1.3. Protein-Protein Interaction (PPI) Network Construction and Network Analyses

**Figure 2:**
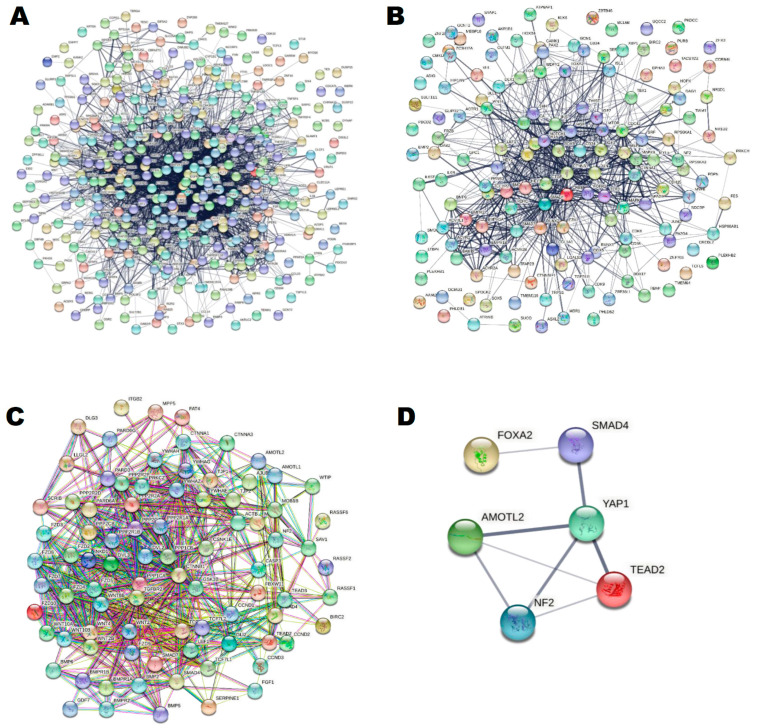
The protein–protein interaction networks that were obtained by using String tool. (**A**) DEGs in cell proliferation gene set. (**B**) DEGs in cell differentiation gene set. (**C**) DEGs in Hippo pathway gene set. (**D**) The filtered selected key hub genes. (http://stringdb.org; version 11.0, accessed on 22 April 2021).

Using the STRING online database and Cytoscape software, the three integrated gene sets of DEGs were mapped into the PPI network complex (Figure 2A–C). In these networks, nodes with a degree >10 were chosen as hub genes (Supplementary Table S2). 

#### 2.1.4. Key Genes for Co-Regulatory Network

Mothers against decapentaplegic homolog 4 *(SMAD4),* Neurofibromin 2 *(NF2),* Angiomotin Like 2 *(AMOTL2),* Forkhead box protein A2 (*FOXA2),* TEA Domain Transcription Factor 2 (*TEAD2)* and Yes-Associated protein 1 (*YAP1)* were selected for the targeted network and validated by several public certified databases to be related to Hippo signaling, cell differentiation/proliferation and NASH (Supplementary Figure S1). These key genes were mapped into the string database for PPI network construction (Figure 2D). 

#### 2.1.5. Retrieval of miRNAs and lncRNAs for Co-Regulatory Network

First, the targeted miRNAs were retrieved from miRWalk 3.0 and there were two miRNAs—mir-650 and miR-1205 could interact with six differentially expressed mRNAs identified above (Supplementary Figure S2). Next, miRWalk2; miRNA: ncRNA target tool, was used to predict the interaction between lncRNAs and miRNAs. Two lncRNAs—RPARP antisense RNA 1 (*RPARP-AS1*) and SRD5A3 Antisense RNA 1 (*SRD5A3-AS1*)—were screened and were interacting with the retrieved miRNAs (Supplementary Figure S3).

Finally, the (mRNAs *YAP1, FOXA2, AMOTL2, TEAD2, SMAD4* and *NF2*)–(miR-650 and miR-1205)–(lncRNAs *SRD5A3-AS1 and RPARP-AS1*) regulatory network was constructed. 

### 2.2. Laboratory Validation of the Constructed RNA Network in Animal Model

#### 2.2.1. Histopathological Evaluation of Liver Tissue Using Different Special Stains

**Figure 3:**
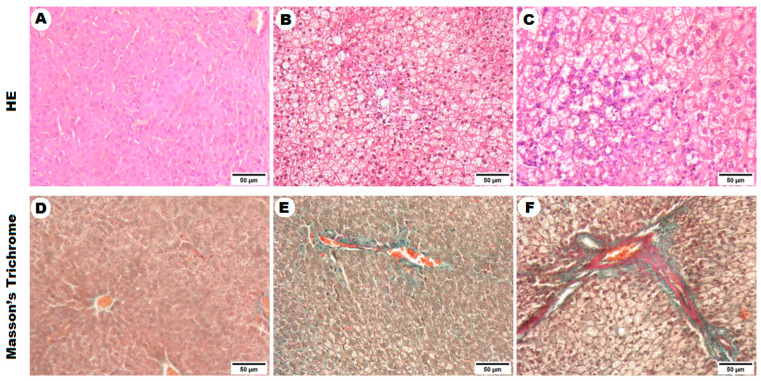
Histopathological changes in liver tissue of the experimental groups using HE and Masson’s Trichrome stains. (**A**,**D**) sections from NC group exposed to normal pellet diet; (**B**,**E**) sections from SS group exposed to HSHF for nine weeks; (**C**,**F**) sections from NASH group exposed to HSHF for 12 weeks (magnification: 200×). NC, Naïve control group; SS, simple steatosis; NASH, non-alcoholic steatohepatitis; HSHF, high sucrose high fat diet; HE, hematoxylin and eosin staining.

Overall, no abnormalities were observed in the liver tissue of the normal control (NC) rats both macroscopically and microscopically. In contrast, livers from rats of the SS and NASH groups were generally enlarged with a yellowish and greasy appearance. Hepatic lobules in normal control rats were intact and clear; no lipid droplets in the liver tissue and the structure of the hepatocytes was normal without inflammatory infiltration or fibrosis (Figure 3A,D). In the NAFLD groups (Figure 3B,C,E), the hepatocytes of rats were swollen, and some cells showed ballooning. The nucleus was squeezed to the side; the varied size and amount of the lipid droplets and lipid vacuoles of both macro- and microvesicular patterns were existent in most hepatocytes. Livers of NASH rats at week 12 developed more severe hepatic inflammation with the formation of fibrous septa (Figure 3F).

#### 2.2.2. Validation of the Constructed Network by Quantitative Reverse Transcription PCR (RT qPCR)

**Figure 4:**
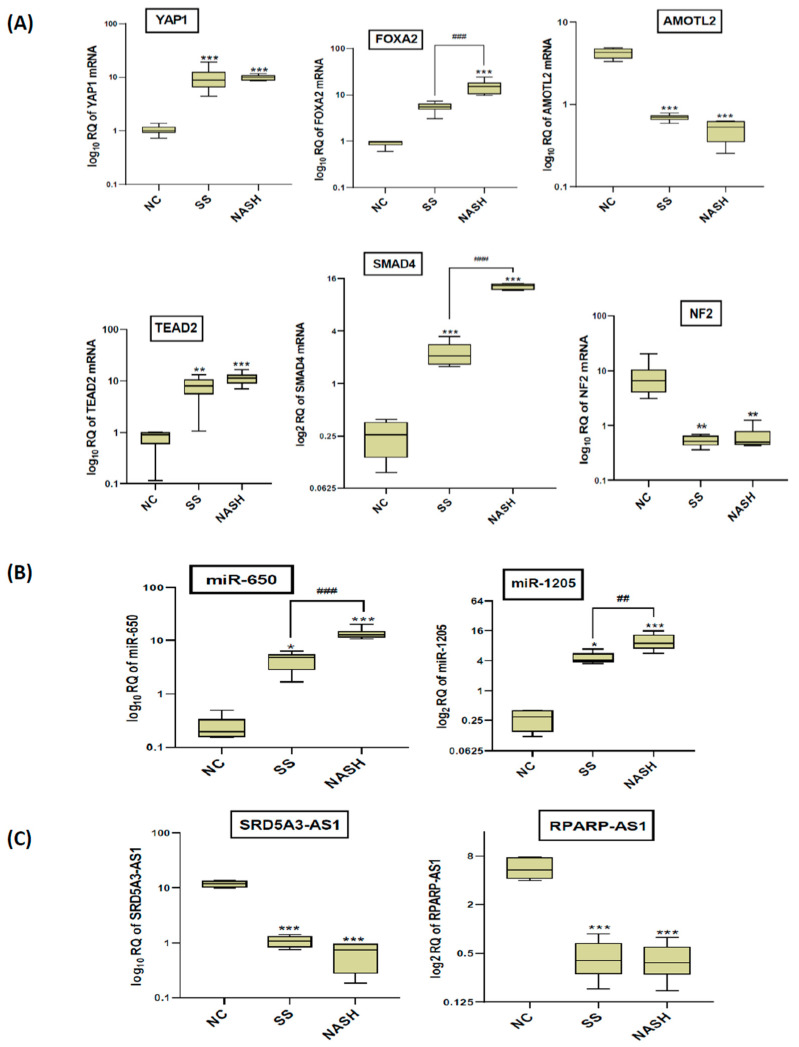
The expression levels of the constructed network members in the experimental groups. (**A**) *YAP1, FOXA2, AMOTL2, TEAD2, SMAD4* and *NF2* m-RNAs. (**B**) miR-650 and miR-1205. (**C**) lncRNAs SRD5A3-AS1 and RPARP-AS1. Data are presented as log fold change; number of animals = six rats/group. *** *p* < 0.001, ** *p* < 0.01 and * *p* < 0.05 compared with NC group; ^###^ *p* < 0.001 and ^##^ *p* < 0.01 significant differences between the two selected groups. RQ, relative quantification; NC, naïve control group; SS, simple steatosis; NASH, nonalcoholic steatohepatitis.

❖ mRNAs expression in the experimental animal models:

The expression levels of *YAP1, FOXA2, AMOTL2, TEAD2, SMAD4* and *NF2* were determined in the liver tissue of the experimental groups (Figure 4A). The results revealed that all the six selected hub genes were differentially expressed in NAFLD animal models. *YAP1* (F value: 17.41, *p* value < 0.001), *TEAD2* (F value: 20.12, *p* value < 0.001) and *SMAD4* (F value: 577.2, *p* value < 0.001) were significantly upregulated in both the NASH and SS groups, compared with the NC group. This significant upregulation was also detected for the FOXA2 level in the NASH group (F value: 32.81, *p* value < 0.001) but not in the SS group, compared to the NC group. On the other hand, *AMOTL2* (F value: 205.9, *p* value <0.001) and *NF2* (F value: 8.489, *p* value < 0.01) appeared to be significantly downregulated in both the NASH and SS groups, compared to the NC group. It was observed that there was no significant variation in the expression of most of the selected genes between the NASH and SS group. However, the expression of *FOXA2* and *SMAD4* significantly increased in the NASH group compared to the SS group.

❖ mi-RNAs expression in the experimental animal models:

As shown in Figure 4B, the relative quantification (RQ) of the *miR-650* (F value: 59.85, *p* value < 0.01) and *miR-1205* (F value: 28.07, *p* value < 0.01) in the liver tissue samples was significantly increased in both the NASH and SS groups, compared to the NC group. In addition, the *miR-650* and miR-1205 were significantly upregulated in the NASH group compared to the SS group.

❖ lnc-RNAs expression in the experimental animal models:

Compared with the NC group, the expression levels of lncRNAs *SRD5A3-AS1* (F value: 304.1, *p* value < 0.001) and *RPARP-AS1*(F value: 59.51, *p* value < 0.001) were significantly decreased in both the NASH and SS groups (Figure 4C). Moreover, there were no significant changes in the expression of *SRD5A3-AS1 and RPARP-AS1* between both forms of NAFLD.

## 3. Discussion

Nonalcoholic fatty liver disease (NAFLD) has become an alarming public health problem. Therefore, it is crucial to study the mechanism and identify molecular targets for diagnosis and treatment [27]. A number of studies have found that the NAFLD pathogenesis is related to a variety of signaling pathways including Hippo pathway [11] with the potential role of miRNAs and lncRNAs, which are crucial in disease development and progression [1]. In the present study, we aimed to perform a comprehensive bioinformatics analysis and retrieve the mRNAs, miRNAs and lncRNAs regulatory network related to Hippo signaling pathway and cell proliferation/differentiation and associated with NAFLD pathogenesis followed by experimental validation of their differential expressions in an NAFLD animal model.

In this study, a total of 8664 DEGs in the GSE33814 dataset for NAFLD were identified. After functional enrichment and protein–protein interaction analyses of these DEGs, three integrated hub gene sets related to cell proliferation (177 genes), cell differentiation (53 genes) and hippo signaling (62 genes) were retrieved. From these hub gene sets, six key genes—*YAP1, FOXA2, AMOTL2, TEAD2*, *SMAD4* and *NF2*—were selected for co-regulatory network construction as a result of their confirmed association through other databases with Hippo signaling pathway, cell proliferation/differentiation and NAFLD pathogenesis. The targeted miRNAs for the co-regulatory network were retrieved and mir-650 and miR-1205 were found to interact with the six differentially expressed mRNAs identified above. Subsequently, two lncRNAs—RPARP-AS1 and SRD5A3-AS1—were screened and were interacting with the retrieved mir-650 and miR-1205. Ultimately, the targeted co-regulatory network was constructed: mRNA (*YAP1, FOXA2, AMOTL2, TEAD2, SMAD4* and *NF2*)–miRNA (*mir-650 and miR-1205*)–lncRNA (*RPARP-AS1 and SRD5A3-AS1*).

The constructed co-regulatory network was utilized on an experimental model of NAFLD for its potential validation of their differential expressions. The two forms of NAFLD—SS and NASH—were induced in rat models by simulating the nutritional habits of modern people using a high-sucrose and high-fat diet for 9 and 12 weeks, respectively [28]. This nutritional model was successfully developed hepatic changes typical of human NAFLD. As reported in the study that was conducted by Masarone et al. [29], liver biopsy was the best choice to differentiate between SS and NASH in T2DM patients and is one of useful tools used to assess the onset of the disease and to predict overall survival. Therefore, in this context, hematoxylin and eosin (HE) and Masson’s Trichrome staining of liver tissue samples were utilized to evaluate the development of NAFLD, and the results showed that fatty liver with hepatic steatosis was observed at week 9. By week 12, steatohepatitis developed, and the development of hepatic fibrosis occurred. 

In the current study, the constructed network was validated to evaluate the role of the Hippo signaling-related genetic network in NAFLD pathogenesis. The importance of Hippo signaling in the liver became an important determinant in liver size control [30]. YAP is one of the main downstream effectors of the Hippo signaling pathway [31]. When it is dephosphorylated, it is translocated into the nucleus and associated with *TEAD*, transcription factor, forming *YAP/TEAD* complex [32]. The transcriptional activation of *YAP/TEAD* complex enhances stem cell self-renewal and promotes cell proliferation, which are important for stimulating liver regeneration. However, aberrant and sustained activation of *YAP* can lead to the formation of malignant tumors [33]. Moreover, it was reported that *TEAD* protein serves an essential role in the regulation of cell proliferation and the developmental process [34,35] and can influence cancer progression through the activation of pro-growth genes transcription [36]. 

Accordingly, in the current study, *YAP1* and *TEAD2* were significantly upregulated in both the SS and NASH group, compared with the NC group. In support to the present findings is the study by Ye et al. [37], who found a decrease in phospho-*YAP* over YAP—i.e., YAP activation, in a mouse model of NASH-precarcinoma. They showed that the fatty acid overload in hepatic cells induced an obesity-associated gene *JCAD*, junctional protein-associated with coronary artery disease. *JCAD* interacted then with large tumor suppressor kinase 1/2 (*LATS1/2),* a core kinase component of the Hippo signaling pathway, and inhibited it to phosphorylate YAP and induce cell proliferation [38]. 

Additionally, *NF2* and angiomotin (*AMOT*) proteins, two upstream components of the Hippo pathway, facilitate *YAP* phosphorylation via *LATS1/2* during cell fate specification and development [39]. *NF2* is a classic tumor suppressor, the loss of which is associated with multiple human tumor types and has been linked to over-proliferation caused by a number of signaling pathways [40].The conditional knock-out of *Nf2* established by Benhamouche et al. [41] led to a reduction in *Lats1/2* phosphorylation and thereby activation of YAP, resulting in hepatic overgrowth and liver tumor development. Zhang et al. [42] reported that *NF2* and *YAP* act antagonistically to each other in the Hippo pathway to regulate liver development and physiology. Additionally, previous findings demonstrated the role of *AMOTl2*, one of *AMOT* family, as a tumor suppressor, where it decreased *YAP* tight junction localization, reduced the accumulation of nuclear *YAP*, and attenuated *YAP* phosphorylation [43]. Han et al. [44] showed that liver-specific deletion of *AMOTL2* in mice resulted in an increase in mouse liver size and activation of *YAP*. All these data can illustrate our findings, where *AMOTL2 and NF2* were significantly downregulated in both the NASH and SS groups, compared to the NC group. 

In the current study, SMAD4 was significantly upregulated in both the NASH and SS groups, compared with the NC group. *SMAD4* is one of mothers against decapentaplegic homolog (*SMAD*) proteins and plays an important role in the occurrence and development of many diseases [45,46]. It was reported to act as a tumor suppressor or oncogenic agent in cancers such as hepatocellular carcinoma [47]. Our results go hand in hand with those of Qin et al. [48], who showed that *SMAD4* protein expression significantly increased in NASH patients than in the control group. Additionally, they reported that deletion of *SMAD4* in NASH mice models showed decreased hepatic steatosis, inflammation, liver cell apoptosis and nonalcoholic fatty liver activity score compared with wild-type mice. These results could be explained by the impact of chronic inflammation to shift hepatocytic *SMAD* phospho-isoform signaling from tumor suppression to carcinogenesis, accelerating liver fibrosis [49]. 

To fulfill the m-RNAs validation, the *FOXA2* level was assessed in the experimental groups. *FOXA2* is one of the transcriptional regulators of the hepatocyte nuclear factor 3 family (*HNF3*). Its members play important roles in regulating cellular proliferation [50] and differentiation [51,52,53]. As *FOXA2* is expressed early during fetal liver development and not expressed in mature hepatocytes or bile duct cells, it might represent an excellent marker of early stage hepatic stem/progenitor cells, HPC [54]. Nobili et al. [55] assessed the role of HPC in pediatric NAFLD comparing histologic specimens isolated from normal controls, fatty livers and NASH. They reported that increased HPC expansion and proliferation was strongly associated with NASH and the stage of fibrosis. Accordingly, this may explain the significant elevation in the level of the *FOXA2* in the NASH group but not in the SS group in the current study. In addition, the mechanism for the regulation of *FOXA2* is perturbed since there is a paradox around its expression in NAFLD. For example, the study by Lake et al. [56] recorded that *FOXA2* mRNA was significantly decreased upon progression to NASH, while the expression of this protein was significantly increased in human NASH livers. Others reported that the levels of FOXA2 mRNA and protein were decreased in hepatocytes from CCl4-induced liver fibrotic mice [57]. In contrast, they also reported that the activated hepatic stellate cells isolated from these fibrotic livers expressed a dramatically higher level of *FOXA2.*

Obviously, several interesting bioinformatics and/or experimental studies demonstrated the potential roles of ncRNAs interaction, including miRNAs and lncRNAs, or their coregulatory interactions with mRNAs in NAFLD pathogenesis [58,59,60,61,62]. miRNAs can regulate gene expression via specific complementary binding to target mRNA, and results in either mRNA degradation or translational suppression [63]. lncRNAs are RNA transcripts (>200 nucleotides in size) without the protein translation capacity but act as a miRNA sponge and can prevent their actions toward the target mRNAs [1]. The role of lncRNAs in inflammation-related diseases, especially NAFLD, was previously discussed in detail in the study by Shabgah et al. [64], who introduced several examples for NAFLD-related lncRNAs and their possible mechanisms contributing to disease development. Utilizing qRT-PCR assay, the present study revealed that the expression of the retrieved *miR-650 and miR-1205 in the liver tissue samples were significantly increased in both the NASH and SS groups compared to the NC group and this increase was more prominent in the NASH group. These results were accompanied by a significant decrease in the expression level of lncRNAs SRD5A3-AS1* and *RPARP-AS1* in both the NASH and SS groups. *miR-650* has been previously reported to be upregulated in different types of cancer, including hepatocellular carcinoma [65], gastric cancer [66] and lung adenocarcinoma [67]. Moreover, *mir-1205* was reported to be associated to induce cell growth and contributes to lung adenocarcinoma and [68] prostate cancer [69]. Regarding lncRNAs, it was retrieved that *RPARP-AS1*(ENSG00000269609) is located on chromosome 10 (25,374 bases), while *SRD5A3-AS1* (ENSG00000249700) is located on chromosome 4 at position q12 (31,877 bases) [70]. 

**Figure 5:**
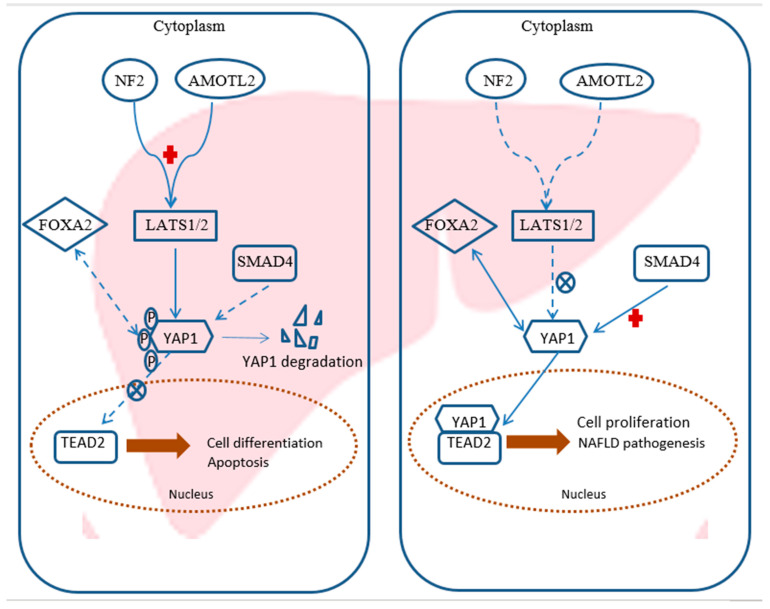
Schematic diagram for the chosen key genes patterns in control and NAFLD model.

Therefore, taken together, our experimental model hypothesized that Hippo signaling-targeted regulatory network (mRNAs *YAP1, FOXA2, AMOTL2, TEAD2, SMAD4* and *NF2*)–miRNA (*mir-650 and miR-1205*)–lncRNA (*RPARP-AS1 and SRD5A3-AS1*) were significant differentially expressed between NAFLD and normal control and play important roles in disease pathogenesis **(**Figure 5). This network may provide a new potential therapeutic target for NAFLD management.

However, some limitations should be noted. This study focused on the differential gene expression of the constructed network without adequate functional analysis; therefore, it will of great importance to carry out further in vitro and in vivo functional studies to validate the current study results. Of note, further large multicentric human studies are urgently required to verify the diagnostic accuracy of the constructed network in NAFLD patients versus healthy control.

## 4. Material and Methods

### 4.1. In-Silico Construction of mRNA-ncRNAs Regulatory Network Using Database Analysis

#### 4.1.1. Raw Data Analysis

The datasets used in the present study were downloaded from the National Center of Biotechnology Information GEO (https://www.ncbi.nlm.nih.gov/geo/, available August 2020) [23]. Datasets including RNA expression profiling for human liver tissues of NAFLD samples were screened. The GSE33814 dataset [71] was identified as it fulfills the following criteria: it was performed on human liver tissue surgical samples and contained whole-gene expression data that differentiate between the NASH, simple steatosis and normal control groups. The GSE33814 dataset contained 12 steatohepatistis samples, 19 steatosis samples, and 13 normal control samples. The platform used to analyze these data was the GPL6884 Illumina HumanWG-6 v3.0 expression beadchip (Illumina, San Diego, CA, USA).

#### 4.1.2. Identification of Differentially Expressed Genes (DEGs)

Microarray data from the GSE33814 dataset were submitted to the online database repository GEO2R (https://www.ncbi.nlm.nih.gov/geo/geo2r/, accessed on 18 April 2021) to identify DEGs among the groups. GEO2R is an interactive web tool based on the R language limma package [72], which can be used to compare two or more groups of samples to identify differential expression in a GEO series. The distribution of the data was visualized using a box-and-whisker plot. The false discovery rate (FDR) is a method of conceptualizing the rate of type I errors in null hypothesis testing when conducting multiple comparisons. GEO2R calculates the FDR automatically. A *p*-value of <0.05 was considered to indicate a statistically significant difference. Probe sets without corresponding gene symbols were removed. 

#### 4.1.3. Functional Enrichment Analyses of the DEGs

Gene ontology (GO) enrichment and pathway analyses of 8664 DEGs were performed using Enrichr (http://amp.pharm.mssm.edu/Enrichr, accessed on 20 April 2021) [73,74]. The biological classification of the DEGs and pathway enrichment analyses were filtered, focusing on the gene sets related to cell differentiation/proliferation and hippo signaling pathway. The species was limited to homo sapiens and the *p*-value ≤ 0.05 was considered statistically significant.

#### 4.1.4. Protein–Protein Interaction Network (PPI) Analysis

The gene sets that were obtained from functional enrichment analyses of the DEGs were imported into the Search Tool for the Retrieval of Interacting Genes (STRING; version 11.0; http://stringdb.org; accessed on 20 April 2021) online database for PPI network construction [75]. PPI pairs with a combined score ≥0.4 were used to construct the PPI networks. The regulatory relationship between genes was visualized using Cytoscape (version 3.7.2) and analyzed through a topological property of the computing network including the degree distribution of the network using the CentiScaPe app [76]. Furthermore, the genes with a degree >10 were defined as hub genes in the regulatory network for the gene set.

#### 4.1.5. Selection of Key Genes for Co-Regulatory Network

From the retrieved hub genes, *SMAD4*, *NF2*, *AMOTL2*, *FOXA2*, *TEAD2*, and *YAP1* were selected as they were strongly linked to NAFLD pathogenesis, cell proliferation /differentiation and hippo signaling pathway and validated either by other public microarray databases—QuickGO (https://www.ebi.ac.uk/QuickGO/), Comparative Toxicogenomics Database (http://ctdbase.org/ accessed on 22 April 2021) and GeneCards (https://www.genecards.org/, accessed on 22 April 2021 )—or by literature reviews [11], [41,43,48,77,78,79,80,81,82]. The final selected genes were then imported into the STRING online database for PPI network construction. 

#### 4.1.6. Prediction of Upstream Key miRNAs and lncRNAs

Interactions between miRNAs and the selected mRNAs were predicted using miRWalk 3.0 (http://mirwalk.umm.uni-heidelberg.de/, accessed on 22 April 2021), which integrated the prediction results of both TargetScan [83] and MiRBase [84]. The interaction between miRNAs and lncRNAs was predicted by using the miRWalk 2.0; miRNA:ncRNA target tool (http://zmf.umm.uni-heidelberg.de/apps/zmf/mirwalk2/mir-mir-self.html, accessed on 22 April 2021). Finally, the mRNAs–miRNAs–lncRNAs regulatory network was established and was validated in an experimental model of NAFLD. 

### 4.2. Experimental Validation of the Constructed Network in NAFLD Animal Model

#### 4.2.1. Experimental Animals and Diets

Studies were performed in 30 male Wistar rats weighing 150 to 180 g and were obtained from the animal House of the Scientific Research Centre at Ain Shams University “MASRI”. Animals were housed in a temperature-controlled (20 ± 2 °C), 12-h light/dark cycle environment with free access to water and normal rat chow. The care and use of animals in this study was under the guidelines approved by the Animal Ethics Committee of Faculty of Medicine, Ain Shams University, Egypt (Ethical Approval Number; FWA000017585, 22 December 2019). The animal model of NAFLD was obtained by feeding the rats a high-sucrose and high-fat (HSHF) diet, which was comprised of 70% normal pellets, 20% lard, 10% sucrose, 1% cholesterol and 0.25% cholic acid [28]. Cholesterol and cholic acid were purchased from Ralin B.V. (Lijinbaan, The Netherlands).

**Figure 6:**
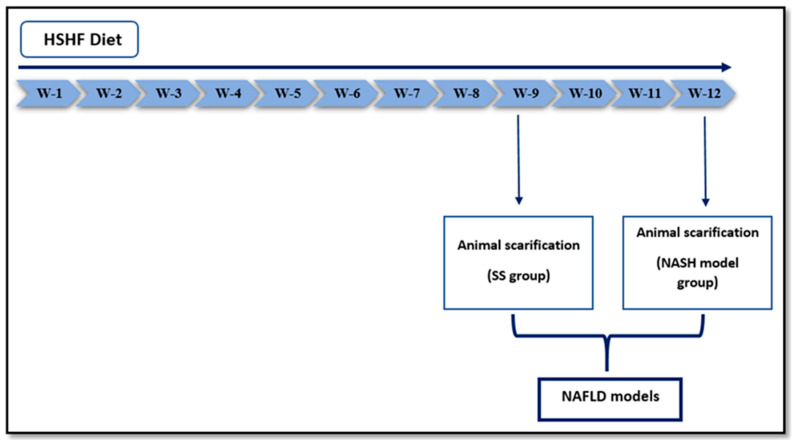
Timeline and study design. HSHF: high sucrose and fat diet; SS: simple steatosis; NASH: nonalcoholic steatohepatitis; NAFLD: nonalcoholic fatty liver disease.

After a week of adaptive feeding on normal rat chow, the rats were randomly divided into three groups of eight rats each: (i) a normal control group (NC), fed a normal pellet diet; (ii) a simple steatosis group (SS), fed an HSHF for nine weeks; and (iii) a NASH model group, fed an HSHF for 12 weeks **(**Figure 6). At the end of the experimental periods, rats were fasted for 12 h and then anesthetized for sacrificing. Liver tissue samples were quickly removed, and part of the tissues were kept in −80 °C for RNAs analysis. A small piece of liver was immediately fixed in 10% neutral-buffered formalin for further histopathological examination.

#### 4.2.2. Tissue Preparation for Histopathological Examination:

Liver tissues fixed in 10% formalin were then dehydrated in ethanol, embedded in paraffin wax, sectioned (5-μm thick) and stained with hematoxylin and eosin (HE) and Masson’s Trichrome stains. 

#### 4.2.3. Extraction of Total RNA (lncRNA, miRNA and mRNA)

The miRNeasy Mini Kit (Cat. No. 217004, Qiagen, Hilden, Germany) was used to extract total RNA from the liver tissue samples as per instructions from the manufacturer. The concentration and purity of total RNA were assessed using Nano Drop 2000 (Thermo Fisher Scientific, Waltham, MA, USA); the purity of the isolated RNAs was 1.8 to 2. The total RNA from the liver tissues was immediately reverse-transcribed into complementary DNA (cDNA) with miScript II RT (Cat. No. 218161, Qiagen) and an RT2 First Strand Kit (Cat. No. 330404, Qiagen) following the manufacturer’s protocol using Thermo Hybaid PCR express (Thermo Fisher Scientific, Waltham, MA, USA).

#### 4.2.4. Real-Time Quantitative Polymerase Chain Reaction (RT-qPCR)

The expressions of mRNAs (*YAP1, FOXA2, AMOTL2, TEAD2, SMAD4*, and *NF2*) in liver tissue samples were estimated using RT2 SYBR- Green ROX qPCR Mastermix (Cat. No. 330522, Qiagen, Germany) and QuantiTect Primer Assays. *miR-650 and miR-1205* expressions in tissue samples were assessed by using miScript SYBR Green PCR Kit (Cat. No. 218073, Qiagen, Germany) and miScript Primer Assays according to the manufacturer’s protocol. The expressions of lncRNAs (RPARP-AS1 and SRD5A3-AS1) in liver tissues were estimated by using RT2 SYBR- Green ROX qPCR Mastermix (Cat. No. 330520, Qiagen, Germany) and RT2 IncRNA qPCR Assay. The Hs_*GAPDH_1*_SG QuantiTect Primer Assay and Hs_*SNORD72_11* miScript Primer Assay were used as the housekeeping genes to normalize the raw data and were then compared with a reference sample. All primer assay reagents used in this study were purchased from Qiagen, Germany and listed in (Supplementary Table S3). RT-qPCR amplification was performed in an Applied Biosystems 7500 FAST Real Time PCR system (Applied Biosystems, Foster City, USA) thermal cycler. The Leviak method was used to examine the relative quantification (RQ) of RNA-based biomarker panel expression, where RQ = 2^−ΔΔ*C*t^ [85].

### 4.3. Statistical Analysis

The GraphPad Prism version 8.0 was used to perform all statistical analyses. The obtained results were expressed as the mean ± SD. The distribution normality of the variables was tested using both the Kolmogorov–Smirnov and Shapiro–Wilk test. For the comparison between groups, analysis of variance (ANOVA) was carried out, followed by Bonferroni’s test. *p* < 0.05 was considered to be statistically significant.

## 5. Conclusions

We used a combined bioinformatics and molecular approach to investigate the role of the Hippo signaling-targeted regulatory network in NAFLD pathogenesis and validation of this network on an NAFLD animal model. The results revealed that mRNA (*YAP1, FOXA2, AMOTL2, TEAD2, SMAD4* and *NF2*)—miRNA (mir-650 and miR-1205)—lncRNA (RPARP-AS1 and SRD5A3-AS1) networks were differentially expressed in NAFLD and strongly associated with disease pathogenesis. This constructed network may be further utilized in future as candidate diagnostic or therapeutic biomarkers for NAFLD management. 

## Figures and Tables

**Table 1:** Functional enrichment analysis after targeted filtration of DEGS related to cell proliferation/differentiation and Hippo signaling.

Identifier	Description	Count in Gene	*p*-Value
**GO:0042127**	Regulation of cell proliferation	371	8.51 × 10^−5^
**GO:0045597**	Positive regulation of cell differentiation	102	0.00850
**GO:0045595**	Regulation of cell differentiation	66	0.013427
**KEGG: hsa04390**	Hippo signaling pathway—Homo sapiens	81	0.025864
**Reactome: R-HSA-2028269**	Signaling by Hippo—Homo sapiens	10	

**Table 2:** The integrated gene sets obtained from enrichment analyses of DEGS.

Cell Proliferation	Cell Differentiation	Hippo Signaling Pathway
*APP, YAP1, CNTF, TES, ADARB1*, *CCAR1, CRKL, TCL1A, CAPNS1*, *FTH1, CYP1B1, SOX9, SOX4, TNS2, TIPIN, RBFOX2, RPL23, CLEC11A, P3H1, FLT3LG, LDOC1, P3H3, ZNF16, SULT2B1, ACE2, SFRP4, TIAM1, EID2, AZGP1, GAREM1*, *SFRP5, CSNK2B, AGTR1, BTK, ATF5, KIF20B, EPHA1, TP53, PLPP1, TSPYL5, BEX4, CTBP1, PRKDC, EIF5A2, PDGFB, PDGFA, TGFA, MST1R, CIAO1, PRDX3, PKHD1, NUAK1, PDCD10, PDGFD, PDGFC, ZNF703, ZNF268, S100A11, ZBTB7C, SLAMF1, MCTS1, JAG2, SRPK2, NGFR, JUN, XBP1, XRCC6, JUND, JAG1, YES1, JUP, XRCC5, INSR, THAP12, FN1, IGF2, LIF, PTK6*, *IFNLR1, IGF1, SMARCA2, ST18, FOSL1, BST1, AGO3, TNFSF4, WNK2, ACER3, SPDYA, CDK10, NOX4, CD27, GNRH1, STRN, GRAP, CALR, CDK13, HRG, ACVRL1, ITK, TENM1, BTG2, CDKN1A, CDKN1B, TGFB1I1, KIF14, AKR1B1, PTPRJ, PTPRK, BRCA1, HOXC10, MECP2, ING5, SHH, HNF4A, TIMP1, IL11, CNOT6L, SYK, IL15, PRMT1, TESC, FSHB, RHOG, EMP2, EMP3, PROX1, SIRT1, CBFA2T3, SIRT2, RHOA, NME1, KIT, RARA, MZB1, SGK3, ARHGEF2, SGK1, CACUL1, GAS8, CCL14, KANK2, XIAP, DERL2, EGFR, FAM129B, MNT, RPS15A, SERTAD1, EPCAM, FRZB, LMNA, PDPN, HSF1, GCNT2, CHRNA10, STX3, TP53I11, CCL23, EGLN3, CDKN2C, CDKN2A, IL34, DAB2IP, VEGFC, AKR1C2, PODN, DHRS2, SLURP1, FABP3, FABP6, PRKRA, CLCF1, DLC1, ABI1, CTNNB1, HSPA1B, CRLF1, FRK, HSPA1A, CD86, ATP8A2, EPO, IRS1, PDCD5, SIRPG, SLA, IRS2, CIB1, FGF1, ETS1, CCND2, ADORA3, PIM2, CAPN1, ARID2, TNFRSF4, IL6R, HIST1H2AC, ENPP7, KRT6A, HIST1H2AB, PDGFRB, PDGFRA, OSR2, RPS9, MAP2K1, IGFBP3, HGF, F2R, CEP131, MATK, ADAM10, LIFR, TSC1, NCCRP1, FOXP3, POU3F2, PGF, CDC25B, ADAM17, HGS, COL4A3, PHIP, STAMBP, ROR2, IL6ST, CSF1R, KMT2D, NOTCH1, SHC1, CUL2, NPR3, TNFRSF11B, FOXO4, ZFP36L1, EFNB2, DPP4, SLC9A3R1, SDCBP, ADGRG1, GRK5, DDRGK1, TMEM127, FGF20, IGFBP7, TNFRSF14, IGFBP6, DRD2, WNT2, CD164, ZBTB17, TFAP2B, TFAP2C, VDR, TNK2, HMGA1, TNFRSF10B, UBE2A, BMX, SMAD6, CHERP, BMP5, BMP2, IL6, CDK6, TMEM115, DLG3, BAMBI, CCPG1, FES, IL7, FGF19, IL9, FGFR1OP, MDM4, NF2, CNOT8, COPS8, IL7R, FGFR3, EIF4G1, FGFR1, BMPR1A, CXCL6, BMPR2, CXCL8, FLT3, CDCA7L, FLT4, HTR2B, PTEN, HMGB2, LAMC2, TCIRG1, FOXM1, DUSP15, PKD2, ADRA1A, CXCL2, CXCL5, RERG, TNFSF13B, RPS4X, SIX4, ADAMTS1, GPER1, NCK2, WDR6, ITGAV, NKX3-1, DIS3L2, KLF10, KLF11, ITGA1, DHPS, NRG1, OPRM1, SSTR1, NGF, ADRA2A, DYNAP, TGFBR2, DUSP22, ZAP70, LCK, TNFRSF25, ITGB1BP1, IRF6, SLC25A5, FXN, BIRC2, TNFRSF21, HDAC4, SRC, HDAC1, LEF1, PLG, DBF4B, PPM1D, DLL1, THBS1, TBRG4, INS, DNAJB2, RAB25, RBBP4, TP53INP1, MXI1, ZNF503, S1PR3, VHL, E2F7, TCFL5, TBX1, NTRK2, TCF7L2, CNBP, NAP1L1, SOD2, PTPN14, MYO16, TBX3, PML, TBX2, CUL4A, TEC, BCL6B, DNAJA2, NEURL1, PTPN6, LTBR*	*NF2, SMAD4, PHLDB1, FOXA2*, *HSP90AB1, PLEKHB2, MEGF10, PLEKHB1, RORC, AKR1B1, CTGF, LGALS3, RPS6KA3, SHH, HEY1, PPP2R1A, TRPS1, RPS6KA1, HEY2, DAG1, DDX17, SERPINF2, EMP2, ISL1, RUNX2, RUNX1, TIAM1, PHLDB2, EPHA3, BIRC2, GCM1, DLX1, DDX5, ZBTB46, SRF, LTBP4, DLL1, KLK6, INS, PURB, PPP2CA, SPOCK2, VHL, ZNF268, TCFL5, TBX1, TFAP2B, CREBBP, JUN, JUND, UQCC2, IGF2, NR1D2, NR1D1, PA2G4, HOPX, SMAD7, CDK9, CDK6, FES, BCL6B, SMOC1, OCIAD1, PDCD2, CAMK1, CD24, BMPR1B, FGFR1, SYAP1, ZC3H12A, SOX9, IL6R, CMKLR1, SOX5, ACVR1, MEF2C, PRKCH, HGF, CTNNBIP1, OLFM1, AGTR1, HOXB4, PRKD1, WDFY2, IL6ST, NOTCH1, PKDCC, TACSTD2, TWIST1, CREBL2, ZFP36L1, SDCBP, ZNF703, WNT4, MSR1, XBP1, SUCO, IGF1, BMP6, COL1A1, BMP2, IL6, DAB2, BAMBI, TMEM119, MYF6, TCF3, EZH2, MYF5, BMPR1A, ACVRL1, BMPR2, TGFB1I1, ATRAID, RBM4, GLIPR2, CD36, HIF1AN, CD34, ADIG, ZHX3, ATP6AP1, PAX2, TGFBR2, CDH15, PPARD, FOXC1, LEF1, CDC42, MAPK9, SULT1E1, FRZB, GPC1, NOCT, PDPN, GCNT2, CTNNA1, AAMDC, SPAG9, TMEM64, MAPK14, ACVR2B, ACVR2A, MTOR, CARM1*, *ASXL2, CTNNB1, ASB4*	*AMOTL2, SMAD4, TEAD2, NF2*, *TJP2, GSK3B, YWHAE, WNT2B, BMPR2, SERPINE1, ITGB2, PPP2R2A, FZD10, FGF1, ACTB, GLI2, PPP1CB, CCND3, RASSF1*, *CCND2, RASSF2, CCND1, PPP2R1B, PPP2R1A, RASSF6, YWHAG, YWHAH, TEAD3, TEAD4, FBXW11, SCRIB, CSNK1E, YWHAZ, TGFBR2, PARD3, AJUBA, BIRC2, LLGL2, TCF7, LEF1, PARD6G, WNT8B, PRKCZ, NKD1, SAV1, PPP2CA, PPP2CB, PARD6A, DVL2, CTNNA1, DVL3, CTNNA3, WNT2, WNT4, WNT10B, TCF7L2, WNT10A, FZD3, TCF7L1, FZD2, FZD5, FZD4, FZD7, FZD9, FZD8, MPP5, WTIP, BMP6, GDF7, SMAD7, BMP5, PPP1CA, MOB1B, MOB1A, BMP2, DLG3, PPP2R2B, ID2, ID1, PPP2R2D, CTNNB1*, *FAT4, BMPR1B, BMPR1A, TJP1, CASP3, AMOTL1*

## Data Availability

The data presented in this manuscript are available upon request from the corresponding authors.

## Supplementary Materials

The following supplementary material are available online at https://www.mdpi.com/article/10.3390/ijms22136770/s1.
