# Supplementary material for: mRNA-miRNA-lncRNA Regulatory Network in Nonalcoholic Fatty Liver Disease
Source: Int J Mol Sci. 2021 Jun 24;22(13):6770. doi: 10.3390/ijms22136770 (PMC8269036; doi:10.3390/ijms22136770)
Supplement: Supplementary file 1 [file ijms-22-06770-s001.zip › Supporting information.pdf]

Supporting information:
Supplementary tables:

Table S3. List of primer assays

Primer assay	
Hs_YAP1_1_SG	(NM_001195044)
Hs_FOXA2_1_SG	(NM_021784)
Hs_AMOTL2_1_SG	(NM_016201)
Hs_TEAD2_1_SG	(NM_001256658)
Hs_SMAD4_1_SG	(NM_005359)
Hs_NF2_1_SG	(NM_000268)
Hs_GAPDH_1_SG	(NM_001256799)
Hs_miR-650_1	(MIMAT0003320)
Hs_miR-1205_1	(MIMAT0005869
Hs_SNORD72_11	(MS00033719)
Human RPARP-AS	(ENST00000473970)
Human SRD5A3-AS	(ENST00000433175)

Supplementary figures:

A. YAP1

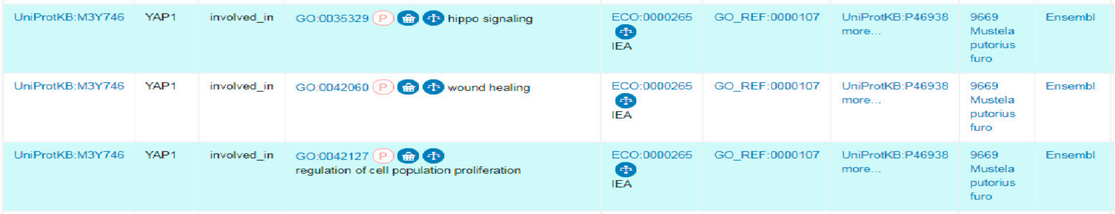

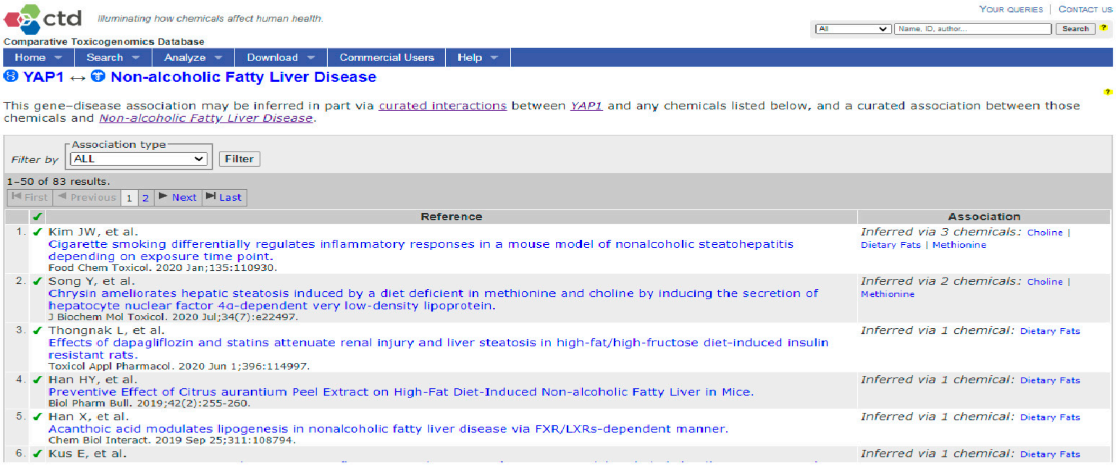

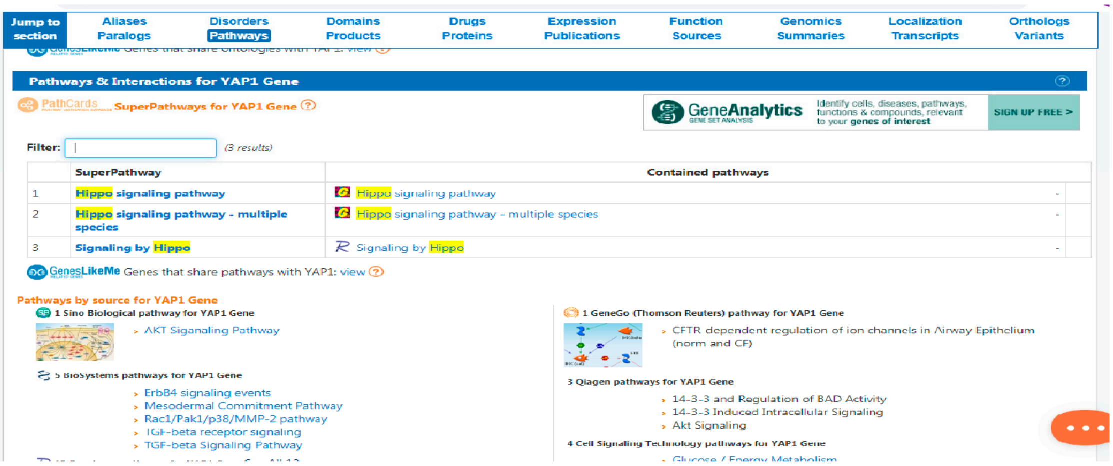

B: SMAD4

UniProtKB:P97471	Smad4	involved_in	GO:0009653 anatomical structure morphogenesis	ECO:0000318 IBA	PMID:21873635	FB:FBgn0011648 more...	10090 Mus musculus	GO_Central
UniProtKB:P97471	Smad4	involved_in	GO:0030154 cell differentiation	ECO:0000318 IBA	PMID:21873635	FB:FBgn0011648 more...	10090 Mus musculus	GO_Central

ctd *Illuminating how chemicals affect human health.*

Comparative Toxicogenomics Database

Your queries | Contact us

All | Name, ID, author... | Search

Home | Search | Analyze | Download | Commercial Users | Help

SMAD4 ↔ Non-alcoholic Fatty Liver Disease

This gene–disease association may be inferred in part via curated interactions between *SMAD4* and any chemicals listed below, and a curated association between those chemicals and *Non-alcoholic Fatty Liver Disease*.

Filter by Association type ALL | Filter

1–50 of 81 results.

First | Previous | 1 | 2 | Next | Last

	✓	Reference	Association
1.	✓	Kim JW, et al. Cigarette smoking differentially regulates inflammatory responses in a mouse model of nonalcoholic steatohepatitis depending on exposure time point. Food Chem Toxicol. 2020 Jan;135:110930.	*Inferred via 1 chemical:* Dietary Fats
2.	✓	Thongnak L, et al. Effects of dapagliflozin and statins attenuate renal injury and liver steatosis in high-fat/high-fructose diet-induced insulin resistant rats. Toxicol Appl Pharmacol. 2020 Jun 1;396:114997.	*Inferred via 1 chemical:* Dietary Fats
3.	✓	Han HY, et al. Preventive Effect of Citrus aurantium Peel Extract on High-Fat Diet-Induced Non-alcoholic Fatty Liver in Mice. Biol Pharm Bull. 2019;42(2):255-260.	*Inferred via 1 chemical:* Dietary Fats
4.	✓	Han X, et al. Acanthoic acid modulates lipogenesis in nonalcoholic fatty liver disease via FXR/LXRs-dependent manner. Chem Biol Interact. 2019 Sep 25;311:108794.	*Inferred via 1 chemical:* Dietary Fats
5.	✓	Kus E, et al. LSEC Fenestrae Are Preserved Despite Pro-inflammatory Phenotype of Liver Sinusoidal Endothelial Cells in Mice on High Fat Diet.	*Inferred via 1 chemical:* Dietary Fats

Jump to section | Aliases Paralogs | Disorders Pathways | Domains Products | Drugs Proteins | Expression Publications | Function Sources | Genomics Summaries | Localization Transcripts | Orthologs Variants

GenesLikeMe Genes that share ontologies with SMAD4: view

Pathways & Interactions for SMAD4 Gene

PathCards SuperPathways for SMAD4 Gene

GeneAnalytics Identify cells, diseases, pathways, functions & compounds, relevant to your genes of interest SIGN UP FREE >

Filter: (1 result)

	SuperPathway	Contained pathways
1	Hippo signaling pathway	Hippo signaling pathway

GenesLikeMe Genes that share pathways with SMAD4: view

C: NF2

UniProtKB:P46662	Nf2	acts_upstream_of_or_within	GO:0046426 negative regulation of receptor signaling pathway via JAK-STAT	ECO:0000266 ISO	GO_REF:0000096	UniProtKB:P35240	10090 Mus musculus	MGI	
UniProtKB:P46662	Nf2	acts_upstream_of_or_within	GO:0045597 positive regulation of cell differentiation	ECO:0000315 IMP	PMID:23863479		10090 Mus musculus	MGI	
UniProtKB:P46662	Nf2	acts_upstream_of_or_within	GO:0045216 cell-cell junction organization	ECO:0000315 IMP	PMID:12695331	MGI:MGI:1857941	10090 Mus musculus	MGI	

ctd *Illuminating how chemicals affect human health.*

Comparative Toxicogenomics Database

Your Queries | Contact us

Home | Search | Analyze | Download | Commercial Users | Help

NF2 ↔ Non-alcoholic Fatty Liver Disease

This gene–disease association may be inferred in part via curated interactions between *NF2* and any chemicals listed below, and a curated association between those chemicals and *Non-alcoholic Fatty Liver Disease*.

Filter by Association type: ALL | Filter

1–50 of 83 results.

First | Previous | 1 | 2 | Next | Last

	✓	Reference	Association
1.	✓	Kim JW, et al.<br>Cigarette smoking differentially regulates inflammatory responses in a mouse model of nonalcoholic steatohepatitis depending on exposure time point.<br>Food Chem Toxicol. 2020 Jan;135:110930.	*Inferred via 1 chemical:* Dietary Fats
2.	✓	Thongnak L, et al.<br>Effects of dapagliflozin and statins attenuate renal injury and liver steatosis in high-fat/high-fructose diet-induced insulin resistant rats.<br>Toxicol Appl Pharmacol. 2020 Jun 1;396:114997.	*Inferred via 1 chemical:* Dietary Fats
3.	✓	Han HY, et al.<br>Preventive Effect of Citrus aurantium Peel Extract on High-Fat Diet-Induced Non-alcoholic Fatty Liver in Mice.<br>Biol Pharm Bull. 2019;42(2):255-260.	*Inferred via 1 chemical:* Dietary Fats
4.	✓	Han X, et al.<br>Acanthoic acid modulates lipogenesis in nonalcoholic fatty liver disease via FXR/LXRs-dependent manner.<br>Chem Biol Interact. 2019 Sep 25;311:108794.	*Inferred via 1 chemical:* Dietary Fats

Jump to section | Aliases Paralogs | Disorders Pathways | Domains Products | Drugs Proteins | Expression Publications | Function Sources | Genomics Summaries | Localization Transcripts | Orthologs Variants

Pathways & Interactions for NF2 Gene

PathCards SuperPathways for NF2 Gene

GeneAnalytics — Identify cells, diseases, pathways, functions & compounds, relevant to your genes of interest — SIGN UP FREE >

Filter: (2 results)

	SuperPathway	Contained pathways	
1	**Hippo signaling pathway**	Hippo signaling pathway	-
2	**Hippo signaling pathway - multiple species**	Hippo signaling pathway - multiple species	-

GenesLikeMe Genes that share pathways with NF2: view

Pathways by source for NF2 Gene

2 BioSystems pathways for NF2 Gene

1 Qiagen pathway for NF2 Gene

D: AMOTL2

UniProtKB:F6SBP4	amotl2	part_of	GO:0031410 cytoplasmic vesicle	ECO:0000318 IBA	PMID:21873635	MGI:MGI:108440 more...	8364 Xenopus tropicalis	GO_Central
UniProtKB:F6SBP4	amotl2	involved_in	GO:0035329 hippo signaling	ECO:0000318 IBA	PMID:21873635	PANTHER:PTN000381812 more...	8364 Xenopus tropicalis	GO_Central

ctd *Illuminating how chemicals affect human health*

Comparative Toxicogenomics Database

Your queries | Contact us

Home | Search | Analyze | Download | Commercial Users | Help

AMOTL2 ↔ Non-alcoholic Fatty Liver Disease

This gene–disease association may be inferred in part via curated interactions between *AMOTL2* and any chemicals listed below, and a curated association between those chemicals and *Non-alcoholic Fatty Liver Disease*.

Filter by Association type: ALL | Filter

1 25 of 25 results.

	✓	Reference	Association
1	✓	Das KP, et al. Perfluoroalkyl acids-induced liver steatosis: Effects on genes controlling lipid homeostasis. Toxicology. 2017 Mar 1;378:37 52.	*Inferred via 1 chemical:* pirinixic acid
2.	✓	Duval C, et al. Chronic Exposure to Low Doses of Dioxin Promotes Liver Fibrosis Development in the C57BL/6J Diet-Induced Obesity Mouse Model. Environ Health Perspect. 2017 Mar;125(3):428 436.	*Inferred via 1 chemical:* Tetrachlorodibenzodioxin
3.	✓	Fader KA, et al. Convergence of hepcidin deficiency, systemic iron overloading, heme accumulation, and REV-ERBα/β activation in aryl hydrocarbon receptor elicited hepatotoxicity. Toxicol Appl Pharmacol. 2017 Apr 15;321:1-17.	*Inferred via 1 chemical:* Tetrachlorodibenzodioxin
4	✓	Lin Y, et al. Downregulation of miR-192 causes hepatic steatosis and lipid accumulation by inducing SREBF1: Novel mechanism for bisphenol A-triggered non-alcoholic fatty liver disease. Biochim Biophys Acta. 2017 Sep;1862(9):869-882.	*Inferred via 1 chemical:* bisphenol A
5.	✓	Ditzel EJ, et al. Altered Hepatic Transport by Fetal Arsenite Exposure in Diet-Induced Fatty Liver Disease. J Biochem Mol Toxicol. 2016 Feb 18.	*Inferred via 1 chemical:* sodium arsenite
6.	✓	Michaut A, et al. A cellular model to study drug induced liver injury in nonalcoholic fatty liver disease: Application to acetaminophen. Toxicol Appl Pharmacol. 2016 Feb 1;292:40-55.	*Inferred via 1 chemical:* Acetaminophen

genecards.org/cgi-bin/carddisp.pl?gene=AMOTL2&keywords=amotl2#pathways_interactions

Jump to section | Aliases Paralogs | Disorders Pathways | Domains Products | Drugs Proteins | Expression Publications | Function Sources | Genomics Summaries | Localization Transcripts | Orthologs Variants

Pathways & Interactions for AMOTL2 Gene

PathCards SuperPathways for AMOTL2 Gene

GeneAnalytics GENE SET ANALYSIS Identify cells, diseases, pathways, functions & compounds, relevant to your genes of interest SIGN UP FREE >

Filter: (1 result)

	SuperPathway	Contained pathways
1	Signaling by Hippo	Signaling by Hippo

GenesLikeMe Genes that share pathways with AMOTL2: view

Pathways by source for AMOTL2 Gene

2 Reactome pathways for AMOTL2 Gene

1 KEGG pathway for AMOTL2 Gene

E: FOXA2

UniProtKB:Q7T1R4	foxa2	enables	GO:0008134 (F) transcription factor binding	ECO:0000256 IEA	GO_REF:0000002	InterPro:IPR013638	8364 Xenopus tropicalis	InterPro
UniProtKB:Q7T1R4	foxa2	enables	GO:0019904 (F) protein domain specific binding	ECO:0000256 IEA	GO_REF:0000002	InterPro:IPR013638	8364 Xenopus tropicalis	InterPro
UniProtKB:Q7T1R4	foxa2	involved_in	GO:0030154 (P) cell differentiation	ECO:0000318 IBA	PMID:21873635	MGI:MGI:1347466 more...	8364 Xenopus tropicalis	GO_Central

Jump to section | Aliases Paralogs | Disorders Pathways | Domains Products | Drugs Proteins | Expression Publications | Function Sources | Genomics Summaries | Localization Transcripts | Orthologs Variants

GPS-Prot Interaction Network for FOXA2

SIGNOR curated interactions for FOXA2 Gene

Signor

Activates: DLK1
Inactivates: OTX2
Is activated by: CTNNB1
Is inactivated by: NANOG, POU5F1, CHUK

Gene Ontology (GO) - Biological Process for FOXA2 Gene

Filter: (2 results)

GO ID	Qualified GO term	Evidence	PubMed IDs
GO:0030154	cell differentiation	IBA	21873635
GO:0071542	dopaminergic neuron differentiation	TAS	24431302

GenesLikeMe Genes that share ontologies with FOXA2: view

ctd *Illuminating how chemicals affect human health.*

Comparative Toxicogenomics Database

Your Queries | Contact Us

All | Name, ID, author... | Search

Home | Search | Analyze | Download | Commercial Users | Help

FOXA2 ↔ Non-alcoholic Fatty Liver Disease

This gene–disease association may be inferred in part via curated interactions between *FOXA2* and any chemicals listed below, and a curated association between those chemicals and *Non-alcoholic Fatty Liver Disease*.

Filter by Association type ALL Filter

1–46 of 46 results.

	✓	Reference	Association
1.	✓	Kim JW, et al.<br>Cigarette smoking differentially regulates inflammatory responses in a mouse model of nonalcoholic steatohepatitis depending on exposure time point.<br>Food Chem Toxicol. 2020 Jan;135:110930.	*Inferred via 2 chemicals:* Choline | Methionine
2.	✓	Song Y, et al.<br>Chrysin ameliorates hepatic steatosis induced by a diet deficient in methionine and choline by inducing the secretion of hepatocyte nuclear factor 4α-dependent very low-density lipoprotein.<br>J Biochem Mol Toxicol. 2020 Jul;34(7):e22497.	*Inferred via 2 chemicals:* Choline | Methionine
3.	✓	Lake AD, et al.<br>Folate receptor-beta expression as a diagnostic target in human & rodent nonalcoholic steatohepatitis.<br>Toxicol Appl Pharmacol. 2019 Apr 1;368:49-54.	*Inferred via 2 chemicals:* Choline | Methionine
4.	✓	Li MH, et al.<br>RLA8-A New and Highly Effective Quadruple PPAR-// and GPR40 Agonist to Reverse Nonalcoholic Steatohepatitis and Fibrosis.<br>J Pharmacol Exp Ther. 2019 Apr;369(1):67-77.	*Inferred via 2 chemicals:* Choline | Methionine
5.	✓	Pogribny IP, et al.<br>Epigenetically mediated inhibition of S-adenosylhomocysteine hydrolase and the associated dysregulation of 1-carbon metabolism in nonalcoholic steatohepatitis and hepatocellular carcinoma.<br>FASEB J. 2018 Mar;32(3):1591-1601.	*Inferred via 4 chemicals:* Choline | Folic Acid | Methionine | Streptozocin
6.	✓	Willebrords J, et al.<br>[illegible]	*Inferred via 1 chemical:* Choline

F: TEAD2

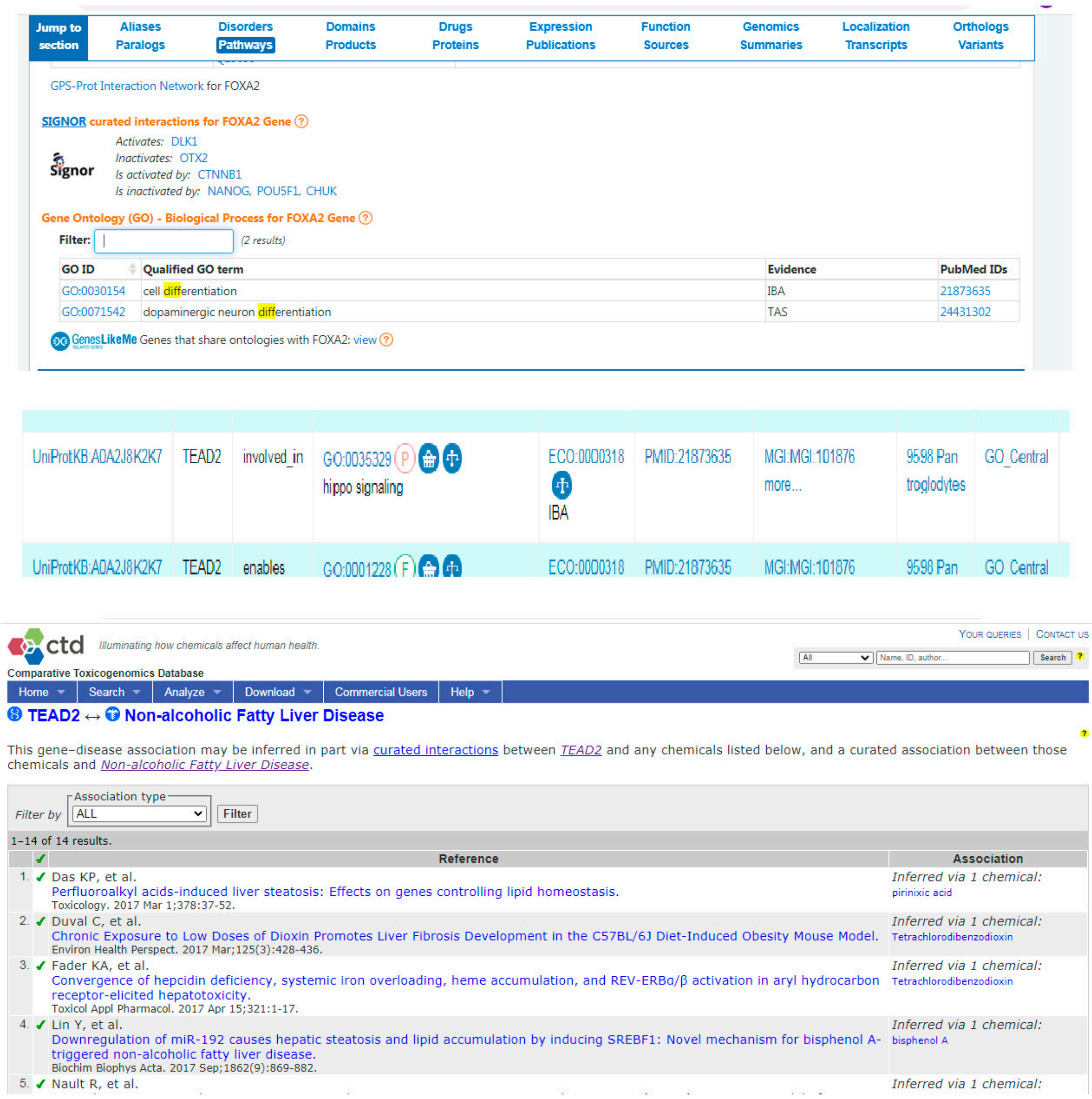

Figures S1. Validation of the relation between key hub genes to NAFLD, cell proliferation/differentiation and Hippo pathway by public databases.

YAP1 & miR-650

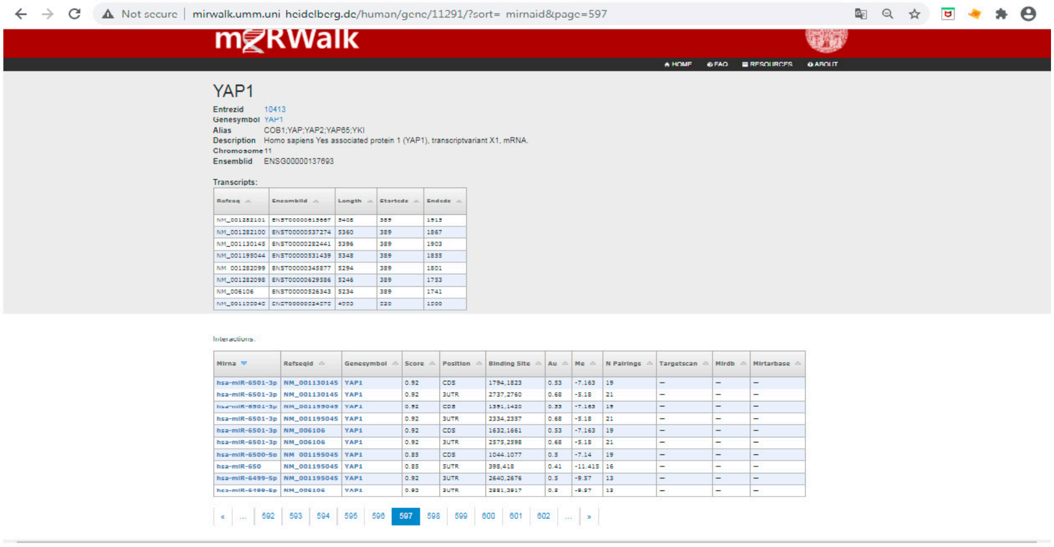

Not secure | mirwalk.umm.uni heidelberg.de/human/gene/11291/?sort= mirnaid&page=597

m🧬RWalk

HOME FAQ RESOURCES ABOUT

YAP1

Entrezid 10413
Genesymbol YAP1
Alias COB1;YAP;YAP2;YAP65;YKI
Description Homo sapiens Yes associated protein 1 (YAP1), transcriptvariant X1, mRNA.
Chromosome 11
Ensemblid ENSG00000137693

Transcripts:

Refseq	Ensemblid	Length	Startcds	Endcds
NM_001282101	ENST00000615667	5408	389	1915
NM_001282100	ENST00000537274	5360	389	1867
NM_001130145	ENST00000282441	5396	389	1903
NM_001195044	ENST00000531439	5348	389	1855
NM 001282099	ENST00000345877	5294	389	1801
NM_001282098	ENST00000629586	5246	389	1753
NM_006106	ENST00000526343	5234	389	1741
NM_001195045	ENST00000524575	4993	520	1500

Interactions:

Mirna	Refseqid	Genesymbol	Score	Position	Binding Site	Au	Me	N Pairings	Targetscan	Mirdb	Mirtarbase
hsa-miR-6501-3p	NM_001130145	YAP1	0.92	CDS	1794,1823	0.53	-7.163	19	–	–	–
hsa-miR-6501-3p	NM_001130145	YAP1	0.92	3UTR	2737,2760	0.68	-5.18	21	–	–	–
hsa-miR-6501-3p	NM_001195045	YAP1	0.92	CDS	1391,1420	0.53	-7.163	19	–	–	–
hsa-miR-6501-3p	NM_001195045	YAP1	0.92	3UTR	2334,2357	0.68	-5.18	21	–	–	–
hsa-miR-6501-3p	NM_006106	YAP1	0.92	CDS	1632,1661	0.53	-7.163	19	–	–	–
hsa-miR-6501-3p	NM_006106	YAP1	0.92	3UTR	2575,2598	0.68	-5.18	21	–	–	–
hsa-miR-6500-5p	NM 001195045	YAP1	0.85	CDS	1044.1077	0.5	-7.14	19	–	–	–
hsa-miR-650	NM_001195045	YAP1	0.85	5UTR	398,418	0.41	-11.415	16	–	–	–
hsa-miR-6499-5p	NM_001195045	YAP1	0.92	3UTR	2640,2676	0.5	-9.57	13	–	–	–
hsa-miR-6499-5p	NM_006106	YAP1	0.92	3UTR	2881,2917	0.5	-9.57	13	–	–	–

« … 592 593 594 595 596 597 598 599 600 601 602 … »

YAP1 & miR-1205

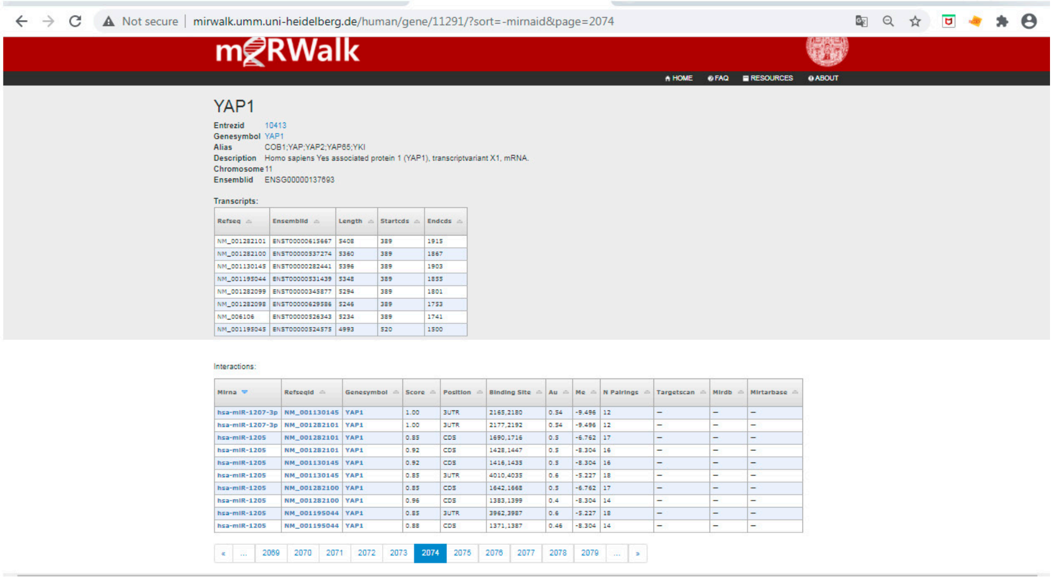

Not secure | mirwalk.umm.uni-heidelberg.de/human/gene/11291/?sort=-mirnaid&page=2074

m🧬RWalk

HOME FAQ RESOURCES ABOUT

YAP1

Entrezid 10413
Genesymbol YAP1
Alias COB1;YAP;YAP2;YAP65;YKI
Description Homo sapiens Yes associated protein 1 (YAP1), transcriptvariant X1, mRNA.
Chromosome 11
Ensemblid ENSG00000137693

Transcripts:

Refseq	Ensemblid	Length	Startcds	Endcds
NM_001282101	ENST00000615667	5408	389	1915
NM_001282100	ENST00000537274	5360	389	1867
NM_001130145	ENST00000282441	5396	389	1903
NM_001195044	ENST00000531439	5348	389	1855
NM_001282099	ENST00000345877	5294	389	1801
NM_001282098	ENST00000629586	5246	389	1753
NM_006106	ENST00000526343	5234	389	1741
NM_001195045	ENST00000524575	4993	520	1500

Interactions:

Mirna	Refseqid	Genesymbol	Score	Position	Binding Site	Au	Me	N Pairings	Targetscan	Mirdb	Mirtarbase
hsa-miR-1207-3p	NM_001130145	YAP1	1.00	3UTR	2165,2180	0.54	-9.496	12	–	–	–
hsa-miR-1207-3p	NM_001282101	YAP1	1.00	3UTR	2177,2192	0.54	-9.496	12	–	–	–
hsa-miR-1205	NM_001282101	YAP1	0.85	CDS	1690,1716	0.5	-6.762	17	–	–	–
hsa-miR-1205	NM_001282101	YAP1	0.92	CDS	1428,1447	0.5	-8.304	16	–	–	–
hsa-miR-1205	NM_001130145	YAP1	0.92	CDS	1416,1435	0.5	-8.304	16	–	–	–
hsa-miR-1205	NM_001130145	YAP1	0.85	3UTR	4010,4035	0.6	-5.227	18	–	–	–
hsa-miR-1205	NM_001282100	YAP1	0.85	CDS	1642,1668	0.5	-6.762	17	–	–	–
hsa-miR-1205	NM_001282100	YAP1	0.96	CDS	1383,1399	0.4	-8.304	14	–	–	–
hsa-miR-1205	NM_001195044	YAP1	0.85	3UTR	3962,3987	0.6	-5.227	18	–	–	–
hsa-miR-1205	NM_001195044	YAP1	0.88	CDS	1371,1387	0.46	-8.304	14	–	–	–

« … 2069 2070 2071 2072 2073 2074 2075 2076 2077 2078 2079 … »

SMAD4 & miR-650

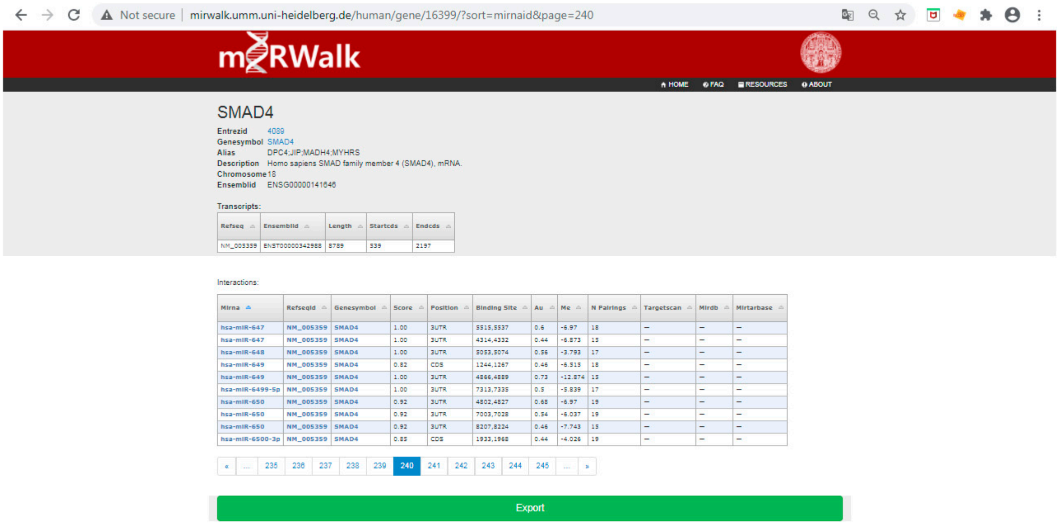

Not secure | mirwalk.umm.uni-heidelberg.de/human/gene/16399/?sort=mirnaid&page=240

miRWalk

HOME FAQ RESOURCES ABOUT

SMAD4

Entrezid 4089
Genesymbol SMAD4
Alias DPC4;JIP;MADH4;MYHRS
Description Homo sapiens SMAD family member 4 (SMAD4), mRNA.
Chromosome 18
Ensemblid ENSG00000141646

Transcripts:

Refseq	Ensemblid	Length	Startcds	Endcds
NM_005359	ENST00000342988	8789	539	2197

Interactions:

Mirna	Refseqid	Genesymbol	Score	Position	Binding Site	Au	Me	N Pairings	Targetscan	Mirdb	Mirtarbase
hsa-miR-647	NM_005359	SMAD4	1.00	3UTR	5515,5537	0.6	-6.97	18	—	—	—
hsa-miR-647	NM_005359	SMAD4	1.00	3UTR	4314,4332	0.44	-6.873	15	—	—	—
hsa-miR-648	NM_005359	SMAD4	1.00	3UTR	5053,5074	0.56	-3.793	17	—	—	—
hsa-miR-649	NM_005359	SMAD4	0.82	CDS	1244,1267	0.46	-6.515	18	—	—	—
hsa-miR-649	NM_005359	SMAD4	1.00	3UTR	4866,4889	0.73	-12.874	15	—	—	—
hsa-miR-6499-5p	NM_005359	SMAD4	1.00	3UTR	7313,7335	0.5	-5.839	17	—	—	—
hsa-miR-650	NM_005359	SMAD4	0.92	3UTR	4802,4827	0.68	-6.97	19	—	—	—
hsa-miR-650	NM_005359	SMAD4	0.92	3UTR	7003,7028	0.54	-6.037	19	—	—	—
hsa-miR-650	NM_005359	SMAD4	0.92	3UTR	8207,8224	0.46	-7.743	15	—	—	—
hsa-miR-6500-3p	NM_005359	SMAD4	0.85	CDS	1933,1968	0.44	-4.026	19	—	—	—

« … 235 236 237 238 239 240 241 242 243 244 245 … »

Export

SMAD4 & miR-1205

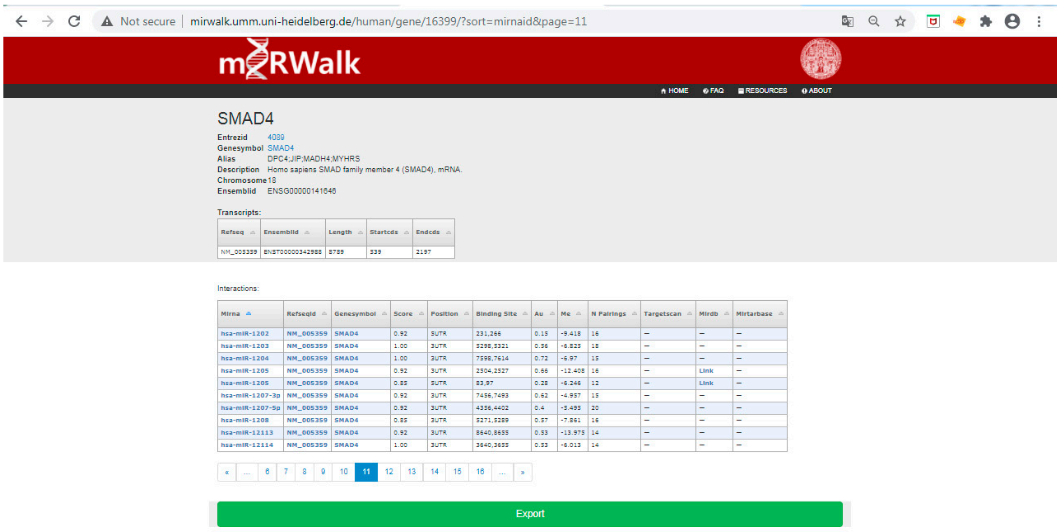

Not secure | mirwalk.umm.uni-heidelberg.de/human/gene/16399/?sort=mirnaid&page=11

miRWalk

HOME FAQ RESOURCES ABOUT

SMAD4

Entrezid 4089
Genesymbol SMAD4
Alias DPC4;JIP;MADH4;MYHRS
Description Homo sapiens SMAD family member 4 (SMAD4), mRNA.
Chromosome 18
Ensemblid ENSG00000141646

Transcripts:

Refseq	Ensemblid	Length	Startcds	Endcds
NM_005359	ENST00000342988	8789	539	2197

Interactions:

Mirna	Refseqid	Genesymbol	Score	Position	Binding Site	Au	Me	N Pairings	Targetscan	Mirdb	Mirtarbase
hsa-miR-1202	NM_005359	SMAD4	0.92	5UTR	231,266	0.15	-9.418	16	—	—	—
hsa-miR-1203	NM_005359	SMAD4	1.00	3UTR	5298,5321	0.56	-6.825	18	—	—	—
hsa-miR-1204	NM_005359	SMAD4	1.00	3UTR	7598,7614	0.72	-6.97	15	—	—	—
hsa-miR-1205	NM_005359	SMAD4	0.92	3UTR	2504,2527	0.66	-12.408	16	—	Link	—
hsa-miR-1205	NM_005359	SMAD4	0.85	5UTR	83,97	0.28	-6.246	12	—	Link	—
hsa-miR-1207-3p	NM_005359	SMAD4	0.92	3UTR	7456,7493	0.62	-4.957	15	—	—	—
hsa-miR-1207-5p	NM_005359	SMAD4	0.92	3UTR	4356,4402	0.4	-5.495	20	—	—	—
hsa-miR-1208	NM_005359	SMAD4	0.85	3UTR	5271,5289	0.57	-7.861	16	—	—	—
hsa-miR-12113	NM_005359	SMAD4	0.92	3UTR	8640,8655	0.53	-13.975	14	—	—	—
hsa-miR-12114	NM_005359	SMAD4	1.00	3UTR	3640,3655	0.53	-6.013	14	—	—	—

« … 6 7 8 9 10 11 12 13 14 15 16 … »

Export

NF2 & miR-650

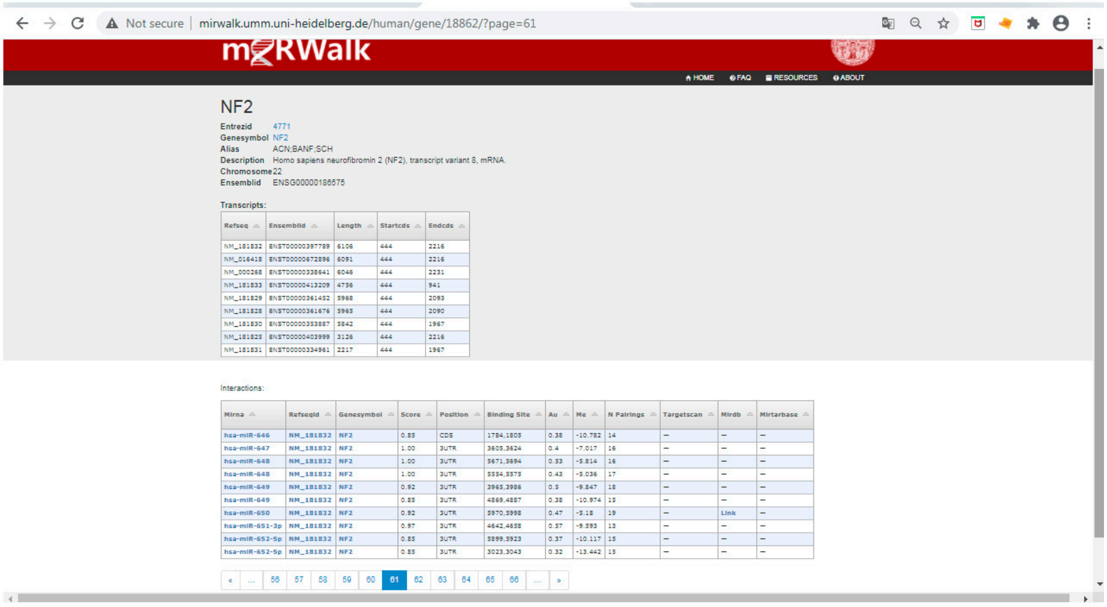

Not secure | mirwalk.umm.uni-heidelberg.de/human/gene/18862/?page=61

miRWalk

HOME FAQ RESOURCES ABOUT

NF2

Entrezid 4771
Genesymbol NF2
Alias ACN;BANF;SCH
Description Homo sapiens neurofibromin 2 (NF2), transcript variant 8, mRNA.
Chromosome 22
Ensemblid ENSG00000186575

Transcripts:

Refseq	Ensemblid	Length	Startcds	Endcds
NM_181832	ENST00000397789	6106	444	2216
NM_016418	ENST00000672896	6091	444	2216
NM_000268	ENST00000338641	6046	444	2231
NM_181833	ENST00000413209	4756	444	941
NM_181829	ENST00000361452	5968	444	2093
NM_181828	ENST00000361676	5965	444	2090
NM_181830	ENST00000353887	5842	444	1967
NM_181825	ENST00000403999	3126	444	2216
NM_181831	ENST00000334961	2217	444	1967

Interactions:

Mirna	Refseqid	Genesymbol	Score	Position	Binding Site	Au	Me	N Pairings	Targetscan	Mirdb	Mirtarbase
hsa-miR-646	NM_181832	NF2	0.85	CDS	1784,1805	0.38	-10.782	14	—	—	—
hsa-miR-647	NM_181832	NF2	1.00	3UTR	3605,3624	0.4	-7.017	16	—	—	—
hsa-miR-648	NM_181832	NF2	1.00	3UTR	5671,5694	0.53	-5.814	16	—	—	—
hsa-miR-648	NM_181832	NF2	1.00	3UTR	5554,5575	0.43	-5.036	17	—	—	—
hsa-miR-649	NM_181832	NF2	0.92	3UTR	3965,3986	0.5	-9.847	18	—	—	—
hsa-miR-649	NM_181832	NF2	0.85	3UTR	4869,4887	0.38	-10.974	15	—	—	—
hsa-miR-650	NM_181832	NF2	0.92	3UTR	5970,5998	0.47	-5.18	19	—	Link	—
hsa-miR-651-3p	NM_181832	NF2	0.97	3UTR	4642,4658	0.57	-9.593	13	—	—	—
hsa-miR-652-5p	NM_181832	NF2	0.85	3UTR	5899,5923	0.37	-10.117	15	—	—	—
hsa-miR-652-5p	NM_181832	NF2	0.85	3UTR	3023,3043	0.32	-13.442	15	—	—	—

« … 56 57 58 59 60 61 62 63 64 65 66 … »

NF2 & miR-1205

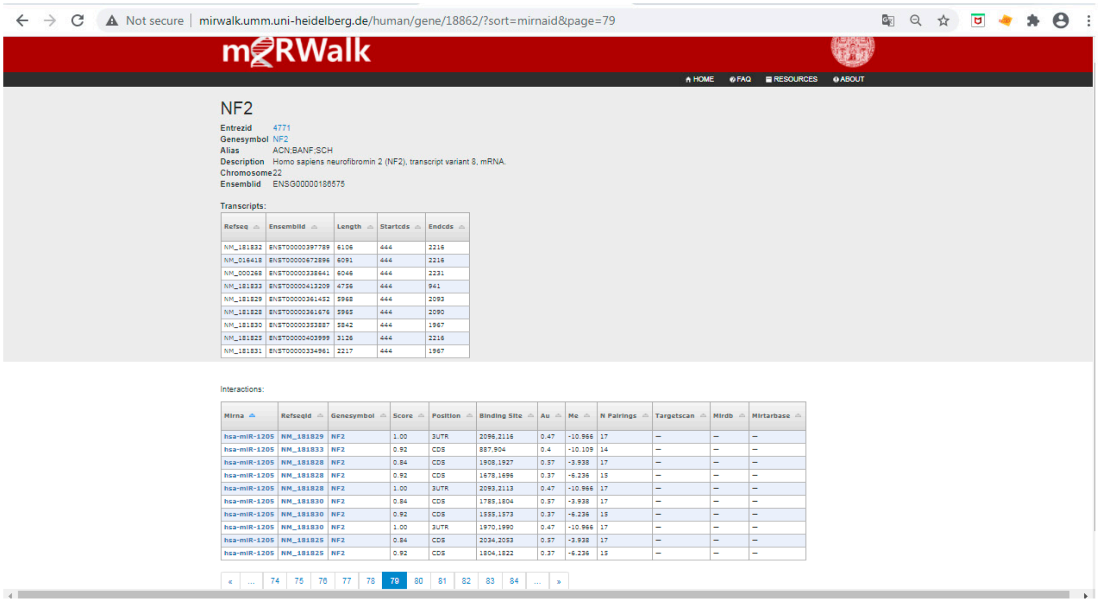

Not secure | mirwalk.umm.uni-heidelberg.de/human/gene/18862/?sort=mirnaid&page=79

miRWalk

HOME FAQ RESOURCES ABOUT

NF2

Entrezid 4771
Genesymbol NF2
Alias ACN;BANF;SCH
Description Homo sapiens neurofibromin 2 (NF2), transcript variant 8, mRNA.
Chromosome 22
Ensemblid ENSG00000186575

Transcripts:

Refseq	Ensemblid	Length	Startcds	Endcds
NM_181832	ENST00000397789	6106	444	2216
NM_016418	ENST00000672896	6091	444	2216
NM_000268	ENST00000338641	6046	444	2231
NM_181833	ENST00000413209	4756	444	941
NM_181829	ENST00000361452	5968	444	2093
NM_181828	ENST00000361676	5965	444	2090
NM_181830	ENST00000353887	5842	444	1967
NM_181825	ENST00000403999	3126	444	2216
NM_181831	ENST00000334961	2217	444	1967

Interactions:

Mirna	Refseqid	Genesymbol	Score	Position	Binding Site	Au	Me	N Pairings	Targetscan	Mirdb	Mirtarbase
hsa-miR-1205	NM_181829	NF2	1.00	3UTR	2096,2116	0.47	-10.966	17	—	—	—
hsa-miR-1205	NM_181833	NF2	0.92	CDS	887,904	0.4	-10.109	14	—	—	—
hsa-miR-1205	NM_181828	NF2	0.84	CDS	1908,1927	0.57	-3.938	17	—	—	—
hsa-miR-1205	NM_181828	NF2	0.92	CDS	1678,1696	0.37	-6.236	15	—	—	—
hsa-miR-1205	NM_181828	NF2	1.00	3UTR	2093,2113	0.47	-10.966	17	—	—	—
hsa-miR-1205	NM_181830	NF2	0.84	CDS	1785,1804	0.57	-3.938	17	—	—	—
hsa-miR-1205	NM_181830	NF2	0.92	CDS	1555,1573	0.37	-6.236	15	—	—	—
hsa-miR-1205	NM_181830	NF2	1.00	3UTR	1970,1990	0.47	-10.966	17	—	—	—
hsa-miR-1205	NM_181825	NF2	0.84	CDS	2034,2053	0.57	-3.938	17	—	—	—
hsa-miR-1205	NM_181825	NF2	0.92	CDS	1804,1822	0.37	-6.236	15	—	—	—

« … 74 75 76 77 78 79 80 81 82 83 84 … »

AMOTL2 & miR-650

[illegible]

mirRWalk

HOME FAQ RESOURCES ABOUT

AMOTL2

Entrezid 51421
Genesymbol AMOTL2
Alias LCCP
Description Homo sapiens angiomotin like 2 (AMOTL2), transcript variant 1,mRNA.
Chromosome 3
Ensemblid ENSG00000114019

Transcripts:

Refseq	Ensemblid	Length	Startcds	Endcds
NM_001278683	ENST00000514516	4991	118	2631
NM_001278685	ENST00000513145	4999	306	2639
NM_016201	ENST00000249883	5008	306	2648
XM_006713654	ENST0000422605	4440	259	2598

Interactions:

Mirna	Refseqid	Genesymbol	Score	Position	Binding Site	Au	Me	N Pairings	Targetscan	Mirdb	Mirtarbase
hsa-miR-6499-3p	NM_001278685	AMOTL2	0.82	CDS	1614,1637	0.31	-6.762	19	–	–	–
hsa-miR-6499-3p	NM_016201	AMOTL2	0.82	CDS	1614,1637	0.31	-6.762	19	–	–	–
hsa-miR-6499-3p	XM_006713654	AMOTL2	0.82	CDS	1567,1590	0.31	-6.762	19	–	–	–
hsa-miR-6499-3p	XM_006713654	AMOTL2	0.85	CDS	679,701	0.25	-5.694	17	–	–	–
hsa-miR-6499-5p	XM_006713654	AMOTL2	0.92	5UTR	168,211	0.25	-9.115	16	–	–	–
hsa-miR-6499-5p	NM_001278685	AMOTL2	0.92	5UTR	238,258	0.25	-9.115	16	–	–	–
hsa-miR-6499-5p	NM_016201	AMOTL2	0.92	5UTR	238,258	0.25	-9.115	16	–	–	–
hsa-miR-650	NM_016201	AMOTL2	0.92	CDS	1041,1065	0.41	-6.283	18	–	–	–
hsa-miR-650	NM_016201	AMOTL2	0.85	CDS	1378,1405	0.37	-5.18	18	–	–	–
hsa-miR-650	NM_001278685	AMOTL2	0.92	CDS	1041,1065	0.41	-6.283	18	–	–	–

« ... 842 843 844 845 846 847 848 849 850 851 852 ... »

Export

AMOTL2 & miR-1205

Not secure | mirwalk.umm.uni-heidelberg.de/human/gene/4092/?sort=mirnaid&page=43

mirRWalk

HOME FAQ RESOURCES ABOUT

AMOTL2

Entrezid 51421
Genesymbol AMOTL2
Alias LCCP
Description Homo sapiens angiomotin like 2 (AMOTL2), transcript variant 1,mRNA.
Chromosome 3
Ensemblid ENSG00000114019

Transcripts:

Refseq	Ensemblid	Length	Startcds	Endcds
NM_001278683	ENST00000514516	4991	118	2631
NM_001278685	ENST00000513145	4999	306	2639
NM_016201	ENST00000249883	5008	306	2648
XM_006713654	ENST0000422605	4440	259	2598

Interactions:

Mirna	Refseqid	Genesymbol	Score	Position	Binding Site	Au	Me	N Pairings	Targetscan	Mirdb	Mirtarbase
hsa-miR-1205	XM_006713654	AMOTL2	0.92	CDS	1355,1379	0.43	-6.97	18	–	–	–
hsa-miR-1205	XM_006713654	AMOTL2	0.85	CDS	677,704	0.28	-3.938	17	–	–	–
hsa-miR-1205	XM_006713654	AMOTL2	0.92	CDS	360,380	0.29	-6.97	17	–	–	–
hsa-miR-1205	NM_001278685	AMOTL2	1.00	CDS	1402,1426	0.43	-6.97	18	–	–	–
hsa-miR-1205	NM_001278685	AMOTL2	0.92	CDS	407,427	0.29	-6.97	17	–	–	–
hsa-miR-1205	NM_001278683	AMOTL2	1.00	CDS	1388,1412	0.43	-6.97	18	–	–	–
hsa-miR-1205	NM_001278683	AMOTL2	0.92	CDS	393,413	0.29	-6.97	17	–	–	–
hsa-miR-1205	NM_016201	AMOTL2	1.00	CDS	1402,1426	0.43	-6.97	18	–	–	–
hsa-miR-1205	NM_016201	AMOTL2	0.92	CDS	407,427	0.29	-6.97	17	–	–	–
hsa-miR-1206	NM_016201	AMOTL2	0.85	3UTR	4098,4124	0.71	-3.938	20	–	–	–

« ... 38 39 40 41 42 43 44 45 46 47 48 ... »

Export

FOXA2 & miR-650

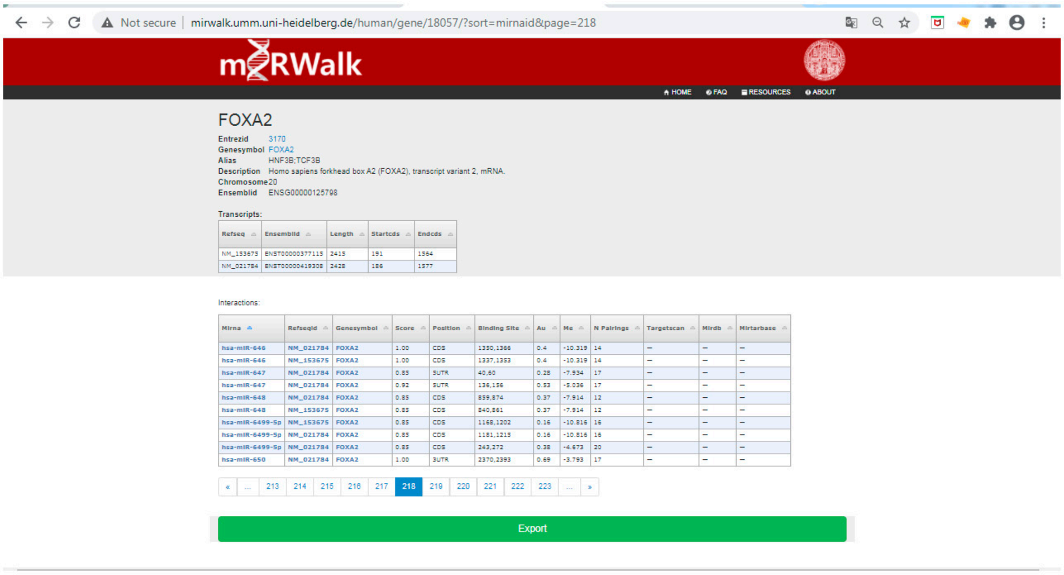

Not secure | mirwalk.umm.uni-heidelberg.de/human/gene/18057/?sort=mirnaid&page=218

miRWalk

HOME FAQ RESOURCES ABOUT

FOXA2

Entrezid 3170
Genesymbol FOXA2
Alias HNF3B;TCF3B
Description Homo sapiens forkhead box A2 (FOXA2), transcript variant 2, mRNA.
Chromosome 20
Ensemblid ENSG00000125798

Transcripts:

Refseq	Ensemblid	Length	Startcds	Endcds
NM_153675	ENST00000377115	2415	191	1564
NM_021784	ENST00000419308	2428	186	1577

Interactions:

Mirna	Refseqid	Genesymbol	Score	Position	Binding Site	Au	Me	N Pairings	Targetscan	Mirdb	Mirtarbase
hsa-miR-646	NM_021784	FOXA2	1.00	CDS	1350,1366	0.4	-10.319	14	–	–	–
hsa-miR-646	NM_153675	FOXA2	1.00	CDS	1337,1353	0.4	-10.319	14	–	–	–
hsa-miR-647	NM_021784	FOXA2	0.85	5UTR	40,60	0.28	-7.934	17	–	–	–
hsa-miR-647	NM_021784	FOXA2	0.92	5UTR	136,156	0.53	-5.036	17	–	–	–
hsa-miR-648	NM_021784	FOXA2	0.85	CDS	859,874	0.37	-7.914	12	–	–	–
hsa-miR-648	NM_153675	FOXA2	0.85	CDS	840,861	0.37	-7.914	12	–	–	–
hsa-miR-6499-5p	NM_153675	FOXA2	0.85	CDS	1168,1202	0.16	-10.816	16	–	–	–
hsa-miR-6499-5p	NM_021784	FOXA2	0.85	CDS	1181,1215	0.16	-10.816	16	–	–	–
hsa-miR-6499-5p	NM_021784	FOXA2	0.85	CDS	243,272	0.38	-4.673	20	–	–	–
hsa-miR-650	NM_021784	FOXA2	1.00	3UTR	2370,2393	0.69	-3.793	17	–	–	–

« ... 213 214 215 216 217 218 219 220 221 222 223 ... »

Export

FOXA2 & miR-1205

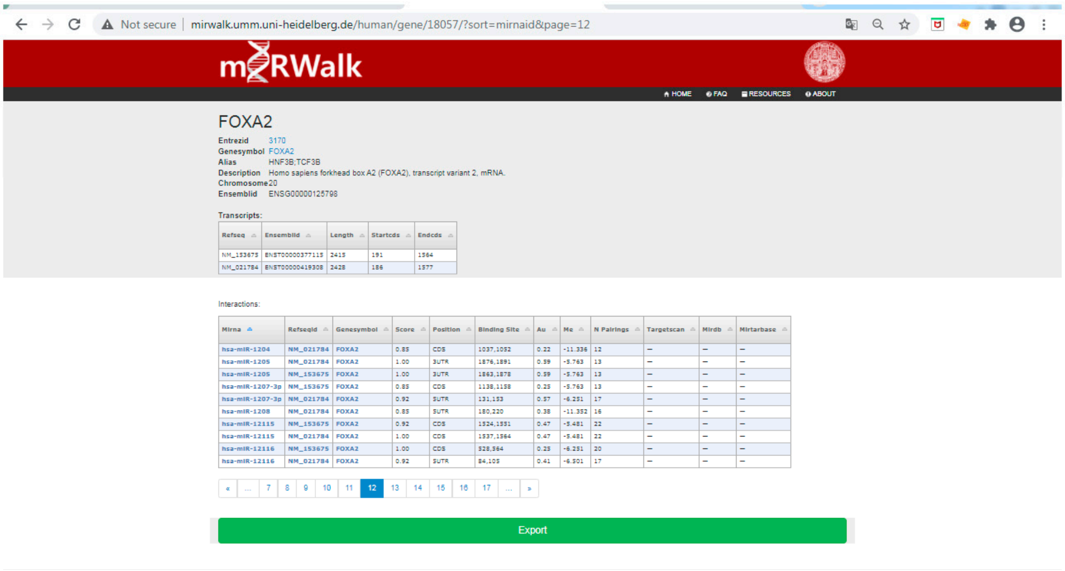

Not secure | mirwalk.umm.uni-heidelberg.de/human/gene/18057/?sort=mirnaid&page=12

miRWalk

HOME FAQ RESOURCES ABOUT

FOXA2

Entrezid 3170
Genesymbol FOXA2
Alias HNF3B;TCF3B
Description Homo sapiens forkhead box A2 (FOXA2), transcript variant 2, mRNA.
Chromosome 20
Ensemblid ENSG00000125798

Transcripts:

Refseq	Ensemblid	Length	Startcds	Endcds
NM_153675	ENST00000377115	2415	191	1564
NM_021784	ENST00000419308	2428	186	1577

Interactions:

Mirna	Refseqid	Genesymbol	Score	Position	Binding Site	Au	Me	N Pairings	Targetscan	Mirdb	Mirtarbase
hsa-miR-1204	NM_021784	FOXA2	0.85	CDS	1037,1052	0.22	-11.336	12	–	–	–
hsa-miR-1205	NM_021784	FOXA2	1.00	3UTR	1876,1891	0.59	-5.763	13	–	–	–
hsa-miR-1205	NM_153675	FOXA2	1.00	3UTR	1863,1878	0.59	-5.763	13	–	–	–
hsa-miR-1207-3p	NM_153675	FOXA2	0.85	CDS	1138,1158	0.25	-5.763	13	–	–	–
hsa-miR-1207-3p	NM_021784	FOXA2	0.92	5UTR	131,153	0.57	-6.251	17	–	–	–
hsa-miR-1208	NM_021784	FOXA2	0.85	5UTR	180,220	0.38	-11.352	16	–	–	–
hsa-miR-12115	NM_153675	FOXA2	0.92	CDS	1524,1551	0.47	-5.481	22	–	–	–
hsa-miR-12115	NM_021784	FOXA2	1.00	CDS	1537,1564	0.47	-5.481	22	–	–	–
hsa-miR-12116	NM_153675	FOXA2	1.00	CDS	528,564	0.25	-6.251	20	–	–	–
hsa-miR-12116	NM_021784	FOXA2	0.92	5UTR	84,105	0.41	-6.501	17	–	–	–

« ... 7 8 9 10 11 12 13 14 15 16 17 ... »

Export

TEAD2 & miR-650

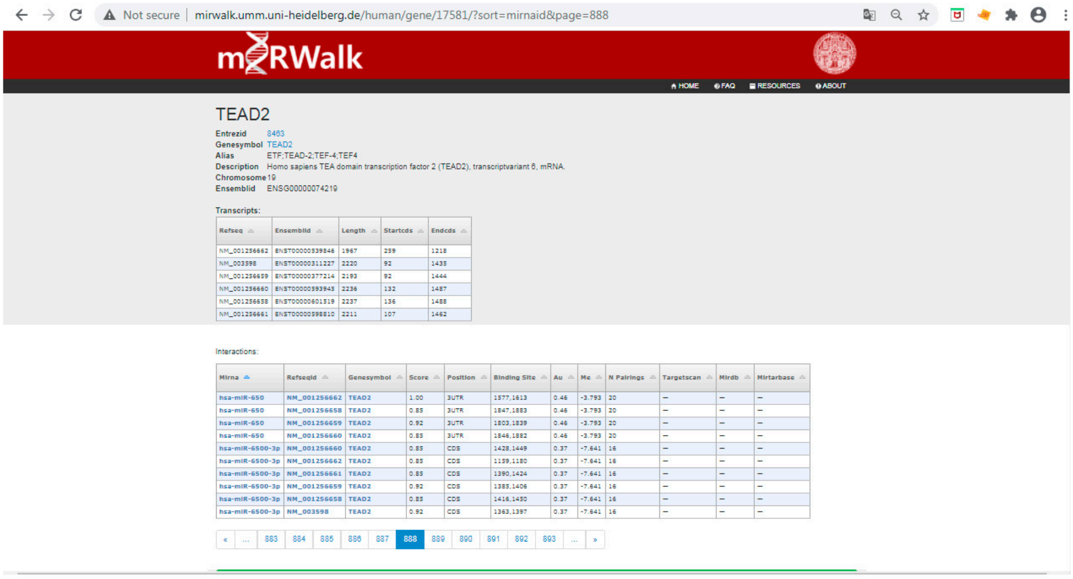

Not secure | mirwalk.umm.uni-heidelberg.de/human/gene/17581/?sort=mirnaid&page=888

mirRWalk

HOME FAQ RESOURCES ABOUT

TEAD2

Entrezid 8463
Genesymbol TEAD2
Alias ETF;TEAD-2;TEF-4;TEF4
Description Homo sapiens TEA domain transcription factor 2 (TEAD2), transcriptvariant 6, mRNA.
Chromosome 19
Ensemblid ENSG00000074219

Transcripts:

Refseq	Ensemblid	Length	Startcds	Endcds
NM_001256662	ENST00000539846	1967	259	1218
NM_003598	ENST00000311227	2220	92	1435
NM_001256659	ENST00000377214	2193	92	1444
NM_001256660	ENST00000593945	2236	132	1487
NM_001256658	ENST00000601519	2237	136	1488
NM_001256661	ENST00000598810	2211	107	1462

Interactions:

Mirna	Refseqid	Genesymbol	Score	Position	Binding Site	Au	Me	N Pairings	Targetscan	Mirdb	Mirtarbase
hsa-miR-650	NM_001256662	TEAD2	1.00	3UTR	1577,1613	0.46	-3.793	20	–	–	–
hsa-miR-650	NM_001256658	TEAD2	0.85	3UTR	1847,1883	0.46	-3.793	20	–	–	–
hsa-miR-650	NM_001256659	TEAD2	0.92	3UTR	1803,1839	0.46	-3.793	20	–	–	–
hsa-miR-650	NM_001256660	TEAD2	0.85	3UTR	1846,1882	0.46	-3.793	20	–	–	–
hsa-miR-6500-3p	NM_001256660	TEAD2	0.85	CDS	1428,1449	0.37	-7.641	16	–	–	–
hsa-miR-6500-3p	NM_001256662	TEAD2	0.85	CDS	1159,1180	0.37	-7.641	16	–	–	–
hsa-miR-6500-3p	NM_001256661	TEAD2	0.85	CDS	1390,1424	0.37	-7.641	16	–	–	–
hsa-miR-6500-3p	NM_001256659	TEAD2	0.92	CDS	1385,1406	0.37	-7.641	16	–	–	–
hsa-miR-6500-3p	NM_001256658	TEAD2	0.85	CDS	1416,1450	0.37	-7.641	16	–	–	–
hsa-miR-6500-3p	NM_003598	TEAD2	0.92	CDS	1363,1397	0.37	-7.641	16	–	–	–

« … 883 884 885 886 887 888 889 890 891 892 893 … »

TEAD2 & miR-1205

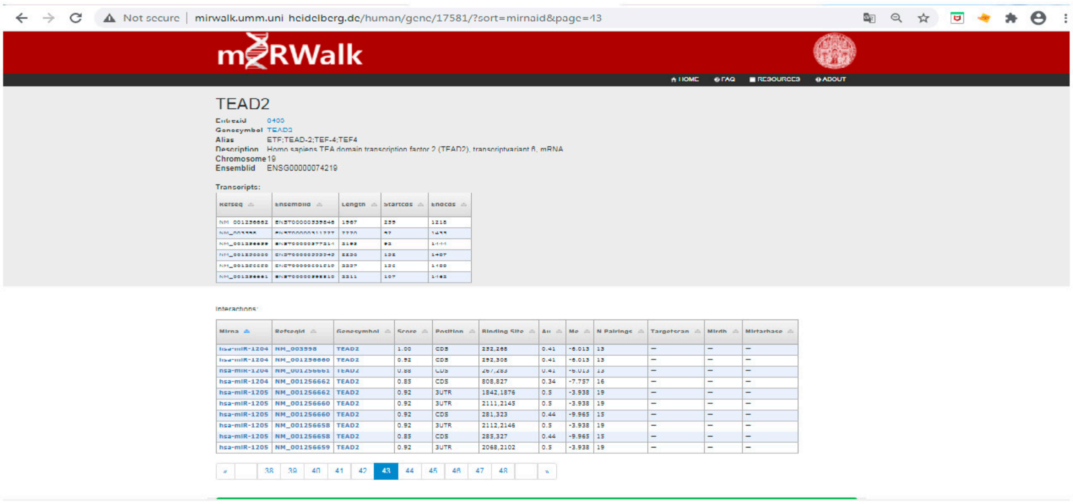

Not secure | mirwalk.umm.uni heidelberg.de/human/gene/17581/?sort=mirnaid&page=43

mirRWalk

HOME FAQ RESOURCES ABOUT

TEAD2

Entrezid [illegible]
Genesymbol TEAD2
Alias ETF;TEAD-2;TEF-4;TEF4
Description Homo sapiens TEA domain transcription factor 2 (TEAD2), transcriptvariant 6, mRNA
Chromosome 19
Ensemblid ENSG00000074219

Transcripts:

Refseq	Ensemblid	Length	Startcds	Endcds
NM_001256662	ENST00000539846	1967	259	1218
[illegible]	[illegible]	[illegible]	[illegible]	[illegible]
[illegible]	[illegible]	[illegible]	[illegible]	[illegible]
[illegible]	[illegible]	[illegible]	[illegible]	[illegible]
[illegible]	[illegible]	[illegible]	[illegible]	[illegible]
[illegible]	[illegible]	[illegible]	107	[illegible]

Interactions:

Mirna	Refseqid	Genesymbol	Score	Position	Binding Site	Au	Me	N Pairings	Targetscan	Mirdb	Mirtarbase
hsa-miR-1204	NM_003598	TEAD2	1.00	CDS	232,268	0.41	-6.013	13	–	–	–
hsa-miR-1204	NM_001256660	TEAD2	0.92	CDS	292,308	0.41	-6.013	13	–	–	–
hsa-miR-1204	NM_001256661	TEAD2	0.88	CDS	267,283	0.41	-6.013	13	–	–	–
hsa-miR-1204	NM_001256662	TEAD2	0.85	CDS	808,827	0.34	-7.757	16	–	–	–
hsa-miR-1205	NM_001256662	TEAD2	0.92	3UTR	1842,1876	0.5	-3.938	19	–	–	–
hsa-miR-1205	NM_001256660	TEAD2	0.92	3UTR	2111,2145	0.5	-3.938	19	–	–	–
hsa-miR-1205	NM_001256660	TEAD2	0.92	CDS	281,323	0.44	-9.965	15	–	–	–
hsa-miR-1205	NM_001256658	TEAD2	0.92	3UTR	2112,2146	0.5	-3.938	19	–	–	–
hsa-miR-1205	NM_001256658	TEAD2	0.85	CDS	285,327	0.44	-9.965	15	–	–	–
hsa-miR-1205	NM_001256659	TEAD2	0.92	3UTR	2068,2102	0.5	-3.938	19	–	–	–

« 38 39 40 41 42 43 44 45 46 47 48 »

Figures S2. Validation of the interaction between the selected m-RNAs and retrieved mi-RNAs from mirwalk.

miR-650 & SRD5A3-AS1

Input form | Web services | Help & Documentation | Bioinformatics Tools FA

```
EMBOSS_001       301 accttgattctactaaaaaaaaaaaaGGATCCTTCAACttcaacaataat     350

hsa-miR-650        1 --------------------------------------------------       0

EMBOSS_001       351 aacacaagggaagTGAAGAAAAACACTTTCTAAGGAAATCTCTCTCTGAG     400

hsa-miR-650        1 --------------------------------------------------       0

EMBOSS_001       401 CTACCATCCTGCTAGGGAGAGCCTGAAAGCAGAATGCTGACTTGACTAGC     450

hsa-miR-650        1 --------------------------------------------------       0

EMBOSS_001       451 TCTGGTCAATGAAAGAAATCACCAGTGGAAAGGCTACCAGGGGGCTTTCT     500

hsa-miR-650        1 --------------------------------------------------       0

EMBOSS_001       501 CAAAAGAGAGGGACAGTAAACTGCAAGATGAACAACTCAATGCCATCCAG     550

hsa-miR-650        1 --------------------------------------------------       0

EMBOSS_001       551 GTCTGGATGAGAAAGAACAGTCGAGAGACTGAGTTTCAGGAGTGGAAACA     600

hsa-miR-650        1 -----------------------------------------AGGAGG---       6
                                                              ||.|||
EMBOSS_001       601 CTCAAAGCATCATCTTCCACTTTCTTCTCCGGCTGCTAGGAAGAAGGGAA     650

hsa-miR-650        7 -----CAG-CGCUCUCAGGAC-----------------------------      21
                          ||| |..:|:.|||||
EMBOSS_001       651 AAACACAGACCTTCTGAGGACCTGCGGAGCAAACCACTGGCTTCAGAAAA     700

hsa-miR-650       22 --------------------------------------------------      21

EMBOSS_001       701 GCAAACAGGGACTTCCCTCTGCGCAGCAGGAAGCAGTTAGCCTAGTCGGA     750
```

miR-650 & RPARP-AS1

Input form | Web services | Help & Documentation | Bioinformatics Tools FAQ

```
#---------------------------------------

hsa-miR-650        1 --------------------------------------------------       0

EMBOSS_001         1 ttttttttttttttttttttgtagagacagggtttcgccatgttgcctag      50

hsa-miR-650        1 --------------------------------------------------       0

EMBOSS_001        51 gctggtctccaacctggtctcctgggctcaagcgatccgcccgcctcggc     100

hsa-miR-650        1 --------------------------------------------------       0

EMBOSS_001       101 ctcccacagtgctgggattccaggcgtgagctaccgcgcccggccTATTT     150

hsa-miR-650        1 --------------------------------------------------       0

EMBOSS_001       151 ACTTTTCTTACTAAGCTGGGGATCACCGTCGCCCTCGGCTTGGCAGGAAG     200

hsa-miR-650        1 ------------------AGGAGG--CAGCGCUCUCAG----GAC-----      21
                                       |.||||  |||..| | |||    |.|
EMBOSS_001       201 GCGGGGGTGCAAGAAGAAAAGAGGTACAGAACACCCAGAGGTGCCCTCGA     250

hsa-miR-650       22 --------------------------------------------------      21

EMBOSS_001       251 TTCCGTCTTGCACTTGCCCTTCTCCCACCGTCCAGCAATAAAGCGAGAGA     300

hsa-miR-650       22 --------------------------------------------------      21

EMBOSS_001       301 AACAAGTGCAGGAAACTGGCCGGCAGTCATGGGAGAAGCCAAAAAGACAG     350

hsa-miR-650       22 --------------------------------------------------      21
```

miR-1205 & SRD5A3-AS1

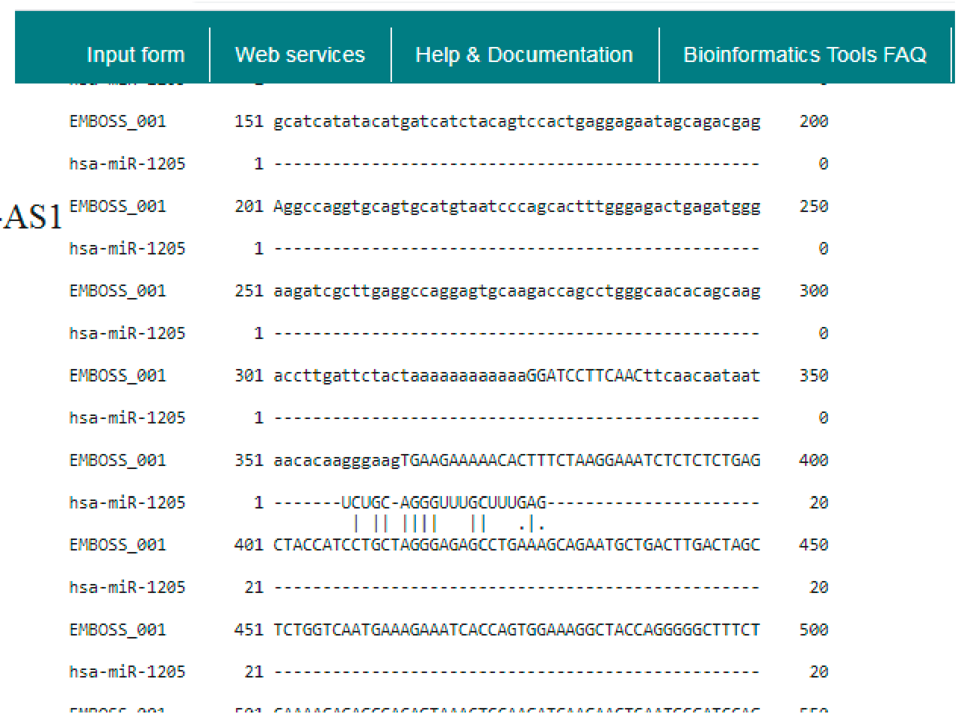

miR-1205 & RPARP-AS1

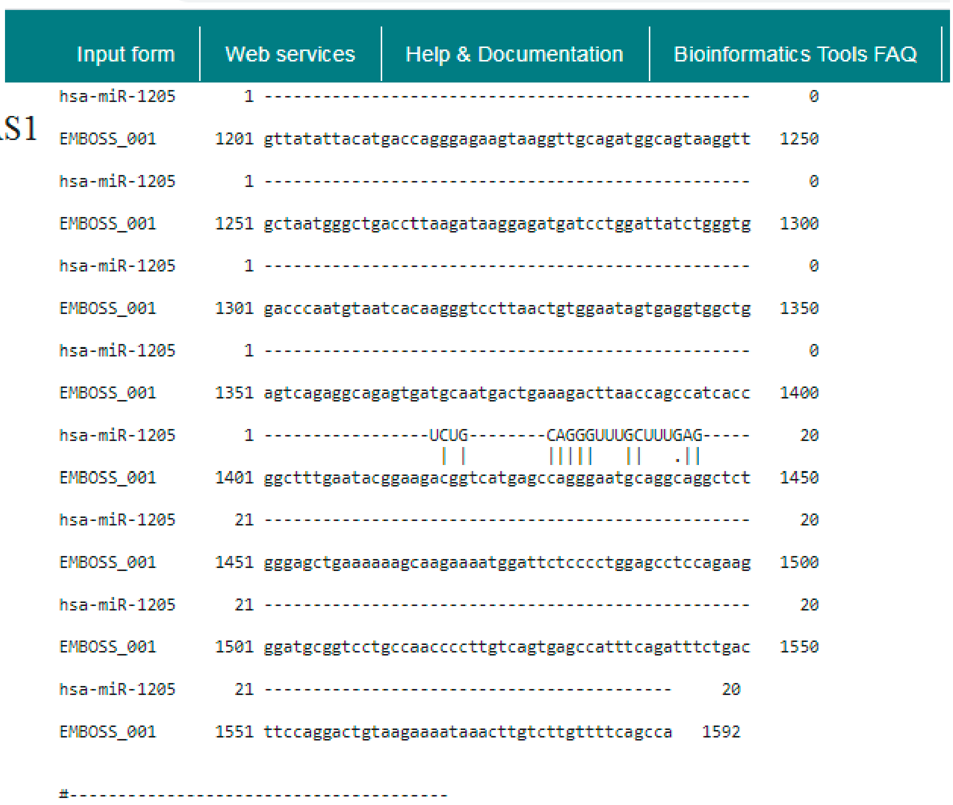

Figures S3. Validation of the interaction between the retrieved mi-RNAs and retrieved lncRNAs.
